# Recognition of G-quadruplex topology through hybrid binding with implications in cancer theranostics

**DOI:** 10.7150/thno.48675

**Published:** 2020-08-20

**Authors:** Yelisetty Venkata Suseela, Pardhasaradhi Satha, N. Arul Murugan, Thimmaiah Govindaraju

**Affiliations:** 1 Bioorganic Chemistry Laboratory, New Chemistry Unit, Jawaharlal Nehru Centre for Advanced Scientific Research, Jakkur P.O., Bengaluru 560064, Karnataka, India.; 2 Department of Theoretical Chemistry and Biology, KTH Royal Institute of Technology, S-106 91, Stockholm, Sweden.

**Keywords:** G-quadruplex, hybrid binding, far-red turn on fluorescence, anti-cancer activity, theranostics

## Abstract

The selective recognition and imaging of oncogene specific G-quadruplex (GQ) structures holds great promise in the development of diagnostic therapy (theranostics) for cancer and has been challenging due to their structural dynamics and diversity. We report selective recognition of GQ by a small molecule through unique hybrid loop stacking and groove binding mode with turn on far-red fluorescence response and anticancer activity demonstrating the potential implications for GQ-targeted cancer theranostics.

**Methods:** Biophysical investigation reveal the turn on far-red emission property of TGP18 for selective recognition of GQ. *In cellulo* studies including DNA damage and oxidative stress evaluation guided us to perform *in vitro* (3D spheroid) and *in vivo* (xenograft mice model) anti-cancer activity, and tumor tissue imaging to assess the theranostic potential of TGP18.

**Results:** Neocuproine-based far-red turn on fluorescence probe TGP18 shows GQ-to-duplex selectivity and specifically recognizes BCL-2 GQ with high affinity through a unique hybrid binding mode involving loop-stacking and groove interactions. Our study reveals that the selective recognition originating from the distinct loop structure of GQ that alters the overall probe interaction and binding affinity. TGP18 binding to anti-apoptotic BCL-2 GQ ablates the pro-survival function and elicit anti-cancer activity by inducing apoptosis in cancer cells. We deciphered that inhibition of BCL-2 transcription synergized with signaling cascade of nucleolar stress, DNA damage and oxidative stress in triggering apoptosis signaling pathway.

**Conclusion:** Intervention of GQ mediated lethality by TGP18 has translated into anti-cancer activity in both *in vitro* 3D spheroid culture and* in vivo* xenograft models of lung and breast cancer with superior efficacy for the former. *In vivo* therapeutic efficacy supplemented with tumor 3D spheroid and tissue imaging potential define the role of TGP18 in GQ-targeted cancer theranostics.

## Introduction

G-quadruplexes (GQs) are non-canonical DNA secondary structures formed by the guanine-rich sequences which regulate a wide range of cellular processes including expression of several oncogenes 1. High throughput sequencing has revealed over 716,310 GQ forming sequences in the human genome and 10,000 of them are identified in the human chromatin using specific antibodies 2-4. Mapping of GQs in the key regulatory regions of the genome including telomeres, oncogene promoters, and untranslated regions of mRNA provide insights into their function and potential therapeutic targets 5. Several GQs in the promoter regions present upstream of proto-oncogenes represent the six hallmarks of cancer 6 including C-MYC, C-KIT and KRAS (self-sufficiency), RB1 (insensitivity), BCL-2 (evasion of apoptosis), VEGFA (angiogenesis), hTERT (limitless replication) and PDGFA (metastasis) and these GQs can be targeted by suitably designed ligands to regulate their expression 5, 7. In cancer cells, stabilization of GQs leads to replication stress and DNA damage accumulation and therefore considered as promising chemotherapeutic target 8, 9.

Ever-increasing interest in exploring the dynamics and functional consequences of GQs in cells requires the development of selective GQ-targeting small molecules. To date, numerous fluorescence and non-fluorescence-based ligands have been reported for selective recognition of GQs over duplex DNA sequences 10-13. Due to subtle structural variations and similar core structure among the multitude of potential GQ-forming sequences, only handful of fluorescence molecules are reported to recognize specific GQ structures 10, 14-17. Interestingly, few non-fluorescence molecules (CX-3543 and CX-5461) have been successful in entering the early phases of clinical trials which underscore the potential of GQ-based therapeutics 18, 19. However, distinct loops and flanking sequences offer possible means to distinguish GQ structures through meticulous probe design to attain the selective binding through specific interactions. Further, fluorescence-based ligands are rarely investigated for therapeutic potential against GQ targets that would uniquely place them as theranostic agents for cancer 20-22. In recent years, theranostic strategies with diagnostic as well as therapeutic capabilities have gained considerable attention, particularly in the development of new generation diagnostic therapy for cancer 23. Theranostic strategy is useful for real-time monitoring of *in vivo* delivery and therapeutic response of the drug candidate. Despite the significant attempts made in nanomedicine to combine therapeutic and diagnostic properties in a single formulation, there are no concerted reports on small molecule theranostics, except for few prodrugs 24-32.

In this context, GQ structures can serve as potential theranostic targets owing to the validation of their incredible role as diagnostic or therapeutic targets. One of the GQ-based theranostic agents reported is BMVC, a carbazole diiodide derivative which suppresses tumor progression and serves as a probe to identify GQ structures with reasonable topology selectivity 20, 22, 33. The wider theranostic application of BMVC is hampered by the high energy excitation (~430 nm) which restricts *in vivo* imaging applications. It is highly desirable to develop fluorescence probes with turn on emission in the far-red to near-infrared (600-800 nm) region of the electromagnetic spectrum for *in cellulo* and *in vivo* bioimaging 10, 34-36. The far-red fluorescence probes exhibit deep tissue penetration and low background interference with minimal damage to the biological samples 10. Undoubtedly, far-red turn on emission and GQ specificity are the key features to be considered while designing theranostic agents. To combine the therapeutic efficacy with excellent bioimaging ability, we disclose neocuproine (phenanthroline)-based molecular probe for specific recognition of a GQ structure with selective fluorescence turn on response in the longer wavelength region, which exhibits propitious *in vivo* anti-cancer activity. Neocuproine was the molecular scaffold of choice as it was shown to recognize and stabilize parallel GQ topologies over duplex, albeit lacked fluorescence response or ability to report any specific GQ topology 37, 38. In view of these shortcomings, our molecular design strategy of neocuproine-based donor-acceptor conjugates offer to deliver promising GQ-targeted theranostics. To the best of our knowledge, leveraging neocuproine-based conjugates to develop GQ-targeted turn on responsive theranostic probe with GQ-topology selectivity has not been reported.

Herein, we demonstrate that one of the neocuproine-based donor-acceptor derivatives TGP18 shows selectivity for GQ over duplex, and specifically recognizes the GQ sequence present upstream of P1 promoter of BCL-2 gene with turn on fluorescence response 39, 40. We investigated the molecular level interactions rendering specific recognition using molecular dynamic simulations and confirmed the results with various biophysical techniques. The B-cell CLL/lymphoma 2 (BCL-2) belongs to the anti-apoptotic gene family and plays crucial role in cell survival functioning as an inhibitor of cell apoptosis, one of the crucial and recognized hallmarks of lung cancer 41, 42. BCL-2 has been found overexpressed in a wide range of human tumors including prostate 43, breast 44, non-small cell lung carcinomas 45, colorectal 46, and B-cell and T-cell lymphomas 47. TGP18 showed transcriptional repression of BCL-2 oncogene causing apoptosis and potential anti-cancer activity in lung cancer xenograft model. Targeting GQ structure resulted in genome instability through the induction of DNA damage and oxidative stress that rendered lung cancer cells sensitive to TGP18.

## Experimental Section

### Chemicals and materials

DMEM, FBS, L-glutamine, penicillin-streptomycin and SYBR Gold (molecular biology grade) were procured from Invitrogen. Caspase-3 assay kit was procured from Thermo Scientific. All single stranded oligonucleotide sequences (Table S1) and the primers for RT-PCR (Table S2) were purchased from Integrated DNA technologies (IDT). DMSO, MTT, Hoechst 33258 and DAPI were obtained from Sigma Aldrich (Merck). All other chemicals were of analytical reagent grade and used without further purification unless otherwise stated. Ultrapure water (double-distilled) obtained from Milli-Q Gradient ultrapure water system (Millipore) and was used in all experiments. ^1^H and ^13^C NMR spectra were recorded on Bruker AV-400 MHz spectrometer with chemical shifts reported as parts per million (*ppm*) (in DMSO-*d*6, tetramethylsilane as an internal standard) at 20 °C. UV-vis absorption and emission spectra were measured in quartz cuvettes of 1 cm path length. HRMS were obtained on Agilent Technologies 6538 UHD Accurate-Mass Q-TOF LC*/*MS spectrometer. HPLC traces were obtained from Shimadzu analytical HPLC.

### Sample preparation

Stock solutions of TGP17, TGP18 and TGP21 were prepared in DMSO in the order of 10^-3^ M and stored at 4 °C. DNA stock solutions were prepared by dissolving oligo samples in double-distilled water in the order of 10^-4^ M. All experiments were carried out using 20 mM PBS containing 100 mM KCl or NaCl at pH 7.4. All oligonucleotides were dissolved in the above-mentioned buffer and heated in water bath at 95 °C for 10 min. The oligonucleotide samples were slowly cooled to room temperature and then stored at 4 °C for 72 h before use for biophysical analysis.

### Synthesis of neocuproine conjugates

Synthesis of starting precursors required for preparation of TGP17, TGP18 and TG21 is given in Scheme S1.

### Synthesis of 7-(diethylamino)-3-(2-(9-methyl-1,10-phenanthrolin-2-yl)vinyl)-2H-chromen-2-one (TGP17)

In a 25 mL round bottom flask 2,9-dimethyl-1,10-phenanthroline **4** (56 mg, 0.27 mmol) was dissolved acetic anhydride (5 mL). 7-(Diethylamino)-2-oxo-2H-chromene-3-carbaldehyde **3** (72 mg, 0.29 mmol) added and the reaction mixture was refluxed for 2 h under nitrogen atmosphere (Scheme S2). After completion of the reaction, solvent was evaporated under reduced pressure. The crude product was purified by column chromatography using dicholoromethane:methanol (DCM:MeOH, from 100:0 to 99:1) as eluant. The product obtained as orange colored solid. Isolated yield 30 mg (25%); ^1^H NMR (400 MHz, CDCl_3_): δ_H_ (ppm) 8.57 (dt, *J* = 8.2, 5.5 Hz, 2H), 8.29 (d, *J* = 9.7 Hz, 2H), 8.17 (s, 1H), 8.13 (s, 2H), 7.93 (d, *J* = 8.2 Hz, 1H), 7.74 (d, *J* = 8.8 Hz, 1H), 7.71 (d, *J* = 5.6 Hz, 1H), 7.00 (dd, *J* = 8.8, 2.5 Hz, 1H), 6.92 (d, *J* = 2.3 Hz, 1H), 3.86 (q, *J* = 7.1 Hz, 4H), 3.36 (s, 3H), 1.66 (t, *J* = 7.1 Hz, 6H).^13^C NMR (100 MHz, CDCl_3_): δ_C_ (ppm) 161.5, 159.3, 156.3, 155.9, 150.8, 150.0, 145.5, 145.4, 139.4, 136.3, 130.1, 129.4, 128.6, 125.7, 125.5, 123.6, 116.5, 109.2, 97.1, 44.9, 25.6, 12.5. HRMS (ESI-TOF, {M + H}): calcd.435.1947 for C_28_H_25_N_3_O_2_, found 436.19801.

### Synthesis of 2-(2-(7-(diethylamino)-2-oxo-2H-chromen-3-yl)vinyl)-1,9-dimethyl-1,10-phenanthrolin-1-ium (TGP18)

In a 25 mL round bottom flask 1,2,9-trimethyl-1,10-phenanthrolin-1-ium **5** (0.1 g, 0.44 mmol) was dissolved in ethanol (5 mL). To the solution of 7-(diethylamino)-2-oxo-2H-chromene-3-carbaldehyde **3** (0.12 g, 0.49 mmol), 2 μL piperidine was added and refluxed for 2 h under nitrogen atmosphere (Scheme S2). Progress of the reaction mixture was monitored by thin layer chromatography (TLC) and after completion of the reaction the solvent was evaporated under reduced pressure. The crude product was purified by column chromatography using DCM: MeOH (98:2) as eluant. The product was obtained as greenish black colored solid. The compound was further purified by reverse phase HPLC using CH_3_CN:water as solvent system. Isolated yield 60 mg (30%); ^1^H NMR (400 MHz, CDCl_3_): δ_H_ (ppm) 9.29 (d, *J* = 8.9 Hz, 1H), 8.92 (s, 1H), 8.88 (d, *J* = 8.8 Hz, 1H), 8.78 (d, *J* = 15.3 Hz, 1H), 8.43 (d, *J* = 15.3 Hz, 1H), 8.29 (d, *J* = 8.3 Hz, 1H), 8.00 (d, *J* = 8.6 Hz, 1H), 7.93 (d, *J* = 8.6 Hz, 1H), 7.65 (d, *J* = 1.6 Hz, 1H), 7.63 (s, 1H), 6.67 (dd, *J* = 9.0, 2.4 Hz, 1H), 6.49 (d, *J* = 2.2 Hz, 1H), 5.08 (s, 3H), 3.49 (q, *J* = 7.1 Hz, 4H), 2.89 (s, 3H), 1.26 (d, *J* = 6.4 Hz, 6H). ^13^C NMR (100 MHz, CDCl_3_): δ_C_ (ppm) 160.6, 158.9, 158.6, 157.2, 152.9, 150.0, 145.3, 143.5, 140.1, 138.4,137.2, 131.9, 130.0, 129.9, 129.2, 125.4, 125.0, 124.1, 117.6, 113.8, 110.2, 109.6, 96.6, 49.1, 45.2, 29.6, 25.4, 12.5. HRMS (ESI-TOF, {M}^+^): calcd.450.2182 for C_29_H_28_N_3_O_2_^+^, found 450.2217.

### Synthesis of 2-(2-(8-hydroxy-1,2,3,5,6,7-hexahydropyrido[3,2,1-ij]quinolin-9-yl)vinyl)-1,9-dimethyl-1,10-phenanthrolin-1-ium iodide (TGP21)

In a 50 mL round bottom flask 1,2,9-trimethyl-1,10-phenanthrolin-1-ium **5** (0.15 g, 0.67 mmol) was dissolved in ethanol (10 mL). 8-Hydroxy-1,2,3,5,6,7-hexahydropyrido [3,2,1-ij] quinoline-9-carbaldehyde **6** (160 mg, 0.74 mmol), catalytic amount of piperidine (10 μL) was added (Scheme S2). The above reaction mixture was refluxed for 3 h. After completion of the reaction, solvent was evaporated under reduced pressure. The crude product was purified by column chromatography using DCM:MeOH (from 100:0 to 97.5:2.5) as eluant, product obtained as blue colored solid. Isolated yield 50 mg (17%); ^1^H NMR (400 MHz, DMSO-*d_6_*): δ_H_ (ppm) 9.38 (s, 1H), 8.52 (ddd, J = 44.8, 35.9, 12.0 Hz, 4H), 8.12 (d, J = 8.5 Hz, 1H), 8.03 (d, J = 8.5 Hz, 1H), 7.77 (d, J = 8.3 Hz, 1H), 7.52 (s, 1H), 7.34 (d, J = 15.0 Hz, 1H), 4.76 (s, 3H), 3.31 - 3.26 (m, 4H), 2.82 (s, 3H), 2.65 (dt, J = 25.8, 6.0 Hz, 4H), 1.87 (dd, J = 11.0, 5.7 Hz, 4H).^13^C NMR (100 MHz, DMSO-*d_6_*): δ_C_ (ppm) 163.4, 162.8, 160.5, 153.7, 144.6, 143.1, 134.4, 133.3, 131.7, 130.2, 120.7, 116.7, 114.0, 111.7, 54.9, 54.2, 53.5, 31.9, 30.2, 26.4, 26.0, 25.5.HRMS (ESI-TOF, {M}^ +^): calcd.422.2232 for C_28_H_25_N_3_O_2_, found 422.2214.

### UV absorption and emission spectroscopy

The UV-vis absorption and emission spectra were recorded on Agilent Technologies Cary series UV-vis-NIR absorbance and Cary eclipse fluorescence spectrophotometers, respectively using 10 mm path-length quartz cuvettes. The absorption spectra were scanned from 230 to 800 nm. Fluorescence emission titrations were carried out in 20 mM PBS (100 mM KCl/NaCl), pH 7.4 using 1 μM of TGP18 with incremental addition of different pre-annealed GQ and duplex DNAs until saturation was reached. The excitation wavelength for TGP18 was fixed at 560 nm and the emission wavelength was scanned from 570 nm to 800 nm. The slits for excitation and emission were set at 5 nm. The normalised changes in fluorescence of TGP18 were recorded and used to calculate the dissociation constant (*K*_D_) value by plotting the change in fluorescence (∆F/∆F_max_) at 640 nm versus increasing concentration of TGP18. The experimental data points obtained were fitted in one site specific binding equation:

∆F/ ∆F_max_ = B_max_. L. (K_D_+L )^-1^ (1)

where ∆F/∆F_max_ = Change in fluorescence, L = GQ concentration and K_D_ = dissociation constant. Similar experiment was performed with duplex DNA, where 1 μM TGP18 was titrated with increasing concentrations of duplex DNA (0-30 μM). The LOD value was calculated by using the equation LOD = K × S_b_/*m*, where K is a numerical factor chosen according to the confidence level desired, S_b_ the standard deviation of the blank measurements (n = 3) and *m* the sensitivity of the calibration curve 48.

### Fluorescence lifetime study

Fluorescence lifetime measurements were performed on a Horiba Delta Flex time-correlated single photon counting (TCSPC) instrument. A 560 nm nano-LED with a pulse repetition rate of 1 MHz was used as the light source. The instrument response function (IRF) was collected using a scatterer (Ludox AS40 colloidal silica). Fluorescence lifetime (λ_exc_ = 560 nm) and gated emission was measured on FLSP920 spectrometer, Edinburgh Instruments equipped with a micro flash lamp (µF2) set-up. From the measured decay traces, the data were fitted with a multi-exponential decay, and χ^2^ was less than 1.1.

### Polyacrylamide gel electrophoresis (PAGE)

PAGE was performed in 1× TBE buffer solution (90 mM tris-boric acid and 2 mM EDTA) using 15% polyacrylamide gel containing 100 mM KCl. Oligonucleotides (1 μM) were loaded on the gel, and electrophoresis was run at 90 V for 1 h at 4 °C. After electrophoresis, the gel was stained using either 100 μM TGP18 in Tris-K^+^ or 1× SYBR Gold, under constant agitation for 15 min, then lightly rinsed with water and visualised using Chemidoc MP imaging system (Biorad). Fluorescence images were acquired with excitation wavelength of 532 nm using the emission filters of 575 nm (for SYBR Gold) and 640 nm (for TGP18). The histogram was generated using Image J software.

### Thermal melting analysis

Thermal melting analysis of all GQs was performed using circular dichroism (CD). CD spectra were recorded on a Jasco 815 spectrometer equipped with a Peltier-type temperature controller (CDF-4265*/*15) under nitrogen atmosphere to avoid water condensation. Scans were performed over wavelength range of 220-500 nm with a speed of 100 nm*/*min, and each spectrum represent an average of three scans with a band width of 1 nm. All measurements were carried out using 10 mm path-length cuvette. 2 μM of each GQ was added to TGP18 at a stoichiometry ratio of 1:1 and incubated for 10 min before spectra were recorded. DNA melting experiments were performed by varying temperature from 20 °C to 90 °C at an interval of 5 °C. All the CD melting experiments were repeated thrice. A blank sample containing 20 mM PBS solution (100 mM KCl, pH = 7.4) was used for baseline correction. For thermal denaturation of duplex (DM7) DNA, the absorption at 260 nm was monitored in the absence or presence of TGP18 over 20-90 °C (1 °C min^-1^, 5 °C intervals) using variable temperature mode in absorption spectroscopy. Melting temperature (T_m_) was calculated from the first derivative of the absorption/temperature curve. Final analysis of the data was carried out by using Origin Pro 8.0.

### Molecular dynamic simulations

The molecular docking was carried out using autodock4 software 49. Structure of the BCL-2 GQ was obtained from the protein databank (reference ID is 2F8U) 40. All three ligands (TGP17, TGP18 and TGP21) were built using molden software and geometry optimised using Gaussian09 software 50 at B3LYP/6-31+G* level of theory 51. Blind docking was carried out and subjected the entire GQ structure for the identification of the binding sites. The centre of the grid box was chosen as the centre of mass of the GQ and the number of grid points were chosen as 130, 130, 110 with a default grid size of 0.375 Å. The Lamarckian genetic algorithm was used to locate various binding sites and binding modes for the ligands within GQ. As many as 500 low energy complex configurations were stored for each ligand:GQ complex. Molecular dynamic (MD) simulations were also carried out to study the stability of TGP18 binding to GQ. Structure from docking was used as the input configuration for MD. The GAFF force-field was used to describe the dispersion interaction, while atomic charges obtained using CHELPG method were used to describe the electrostatic interaction. In particular B3LYP/6-31+G* level of theory was used, and the calculation was performed in water as a solvent described using polarizable continuum model. For the GQ, FF99SB force-field was used and water solvent is described using TIP3P force-field. Suitable number of counter ions were added to neutralise the system and the simulation box was solvated with as many as 15000 water molecules. The simulations were carried out at 300 K and 1 atm pressure. 1 fs was used as the time step to integrate equation of motion and an equilibration run of 5 ns was carried out. The final production run in isothermal-isobaric ensemble was carried out for 50 ns. The binding free energy calculations using MM-GBSA approach were carried out for 2500 configurations corresponding to last 5 ns. A similar procedure was adopted to estimate the binding free energy of TGP18 with other GQs, and TGP17 and TGP21 with BCL-2 GQ. Finally, RAMD simulation was carried to study the egression pathway of TGP18 from BCL-2 GQ in its binding Mode I.

### Cell culture

Human breast adenocarcinoma cell lines (MCF-7 and MDA-MB-231) (ATCC), lung adenocarcinoma cell line (A549) (ATCC), and cervical adenocarcinoma cell line (HeLa) (ATCC) and human embryonic kidney cells (HEK293T) were separately cultured in complete DMEM (Invitrogen) and supplemented with 10% (v/v) FBS, 2 mM L-glutamine, 50 μg/mL gentamycin, 1% Pen-Strep in a fully humidified CO_2_ incubator at 37 °C and 5% CO_2_.

### Cell viability

MTT assay was performed with different cancer cell lines to evaluate the selective anti-proliferative properties of TGP18. MCF-7, MDAMB231, HeLa, A549 and HEK293T cells were sub-cultured into 96-well microtiter plates at a density of 1×10^4^ cells/well in 100 μL of respective culture media treated with an increasing concentration gradient (0-50 µM) of TGP18 for 24 h. MTT solution (15 μL, 5 mg/mL) was added to each well and incubated for 3 h at 37 °C. The precipitated formazan crystals were dissolved with 100 μL DMSO after discarding the cell culture media. Absorbance was recorded at 570 nm using microplate reader (Spectramax i3x, Molecular Devices) having a reference wavelength of 690 nm. The experiments were repeated three time independently. IC_50_ (half-maximum inhibitory concentration) values were obtained from fitting the data in four parametric logistic equation using GraphPad Prism 6.0.

### Quantitative RT-PCR

Real time polymerase chain reaction (RT-PCR) was performed to investigate the transcription inhibitory role of TGP18 in C-MYC, KRAS, BCL-2 and VEGF oncogenes having putative quadruplexes in their promoters. A549 cells were sub-cultured into 6-well microtiter plates at a density of 1 × 10^6^ cells per well and treated with different concentrations (1, 5 and 10 µM) of TGP18 for 24 h. Total RNA was isolated from both untreated and treated cells using TRIzol® method (Invitrogen) as per manufacturer's protocol. Total RNA (1 μg, from both untreated and treated) was processed for cDNA synthesis and reverse-transcribed using OneScript^®^ cDNA Synthesis SuperMix (Abcam) following manufacturer's protocol. Real time PCR was carried out using Maxima SYBR Green/ROX qPCR Master Mix (2X) (Thermo Scientific) as per manufacturer's protocol. The housekeeping gene GAPDH was used as an internal control to normalize the variability in gene expression level. PCR primers were designed using Primer-BLAST, NCBI, and analyzed in OligoAnalyser 3.1-IDT (Table S2).

### Cell cycle analysis

A549 cells (1 × 10^6^) per 60 mm petri dish were either untreated (DMSO) or treated with various concentrations of TGP18 (1, 2, 5 and 10 μM) for 24 h. Cells were then trypsinized and collected by centrifugation at 300g for 5 min, and resuspended in PBS containing 10 μg/mL DAPI and 10 μg/mL RNaseA. After incubation for 30 min in dark at 37 °C, cells were analyzed for DNA content using a FACS flow cytometer (BD Biosciences). Cell distribution among cell cycle phases and the percentage of apoptotic cells were evaluated using Cell-Quest Pro software (BD).

### Caspase-3 activity assay

Caspase-3 assay was performed using Enzchek Caspase-3 assay kit following manufacturer's protocol. Lysates prepared from A549 cells were either untreated (DMSO) or treated with TGP18 (5 and 10 μM) and camptothecin (10 μM) for 24 h. Cell lysates were incubated with the Z-DEVD-R110, caspase-3 substrate alone or with the substrate and Z-DEVD-R110 inhibitor (right panel) and analyzed by spectrofluorometry (excitation and emission wavelengths of 496 nm and 520 nm, respectively). Fluorescence response measured was quantified to analyze caspase-3 activity in apoptotic cell lysates.

### Confocal microscopy

Cellular localization of TGP18 was monitored by live and fixed cell imaging. A549 cells grown on confocal dish were incubated with TGP18 (300 nM) for 1 h at 37 °C. For fixed cells, A549 cells were treated with 4% (wt/vol) paraformaldehyde for 10 min and rinsed twice with PBS before incubation with TGP18. After incubation, cells were washed with PBS three times to remove the excess ligand and bathed in DMEM (2 mL) before imaging. Cell nuclei were stained with Hoechst 33258 (8 μg/mL) and DAPI (300 nM) for live and fixed cells, respectively. Localization of TGP18 was viewed under confocal fluorescence microscope (Olympus FV3000).

### Immunofluorescence microscopy

A549 cells were plated in 35 mm glass-bottomed culture dishes (Genetix Biotech) and cultured overnight. The cells were then untreated (DMSO) or treated with TGP18 (0.5, 1 and 5 μM) or PDS (10 μM) for 24 h. As a control, A549 cells were exposed to UV light for 1 h in DMEM media with low serum percentage. After the respective treatment, cells were fixed in 4% (wt/vol) paraformaldehyde at room temperature for 10 min and washed thrice with PBS. The cells were then permeabilised with 0.1% Triton X-100 in PBS for 5 min and blocked with 4% BSA in PBS for 30 min. Fixed cells were then incubated overnight at 4 °C with primary antibodies: rabbit anti-NPM1 antibody (1:50 dilution, Cell Signaling Technology); rabbit anti-Nrf2 antibody (1:500, Invitrogen); mouse anti-γ-H2AX antibody (1:100, Invitrogen) in blocking solution. Cells were washed three times in PBS and incubated for 1 h with secondary antibodies: goat anti-rabbit-Alexa 488; goat anti-mouse-Alexa 488 (1:1000) (Molecular Probes, Invitrogen) in blocking solution. Cells were then washed twice in PBS and stained with DAPI for 2 min. Imaging was performed using a 480 nm and a 560 nm laser connected to a Fluoview confocal microscope (Olympus FV3000) with a 60× numerical aperture 1.4 lens and the data was analyzed using cellsens software (Olympus).

### Hemolytic assay

TGP18 was assayed on human erythrocytes (O blood group) for hemolytic activity. The human blood samples were obtained from healthy volunteers. The blood was centrifuged at 5,000 rpm for 5 min and subjected to repeated washing with sterile PBS to remove plasma. Suspension of human erythrocytes (2%) in sterile PBS were treated with different concentrations of TGP18. After 30 min incubation at room temperature, cells were centrifuged, and the supernatant was used to measure the liberated hemoglobin by monitoring the absorbance at 418 nm. Two controls were prepared without TGP18, negative control received sterile PBS, while positive control received 0.1% Triton X-100. The average value was calculated from triplicate assays. Hemolysis percentage for each sample was calculated by dividing sample absorbance with positive control absorbance (complete hemolysis) multiplied by 100.

### *In vivo* pharmacokinetic studies of TGP18

All animal experiments were performed in accordance with the guidelines of the Committee for the Purpose of Control and Supervision of Experiments on Animals (CPCSEA), India and were approved by the Institutional Animal Ethics Committee (IAEC) of Anthem Biosciences, Bengaluru, India. The maximum tolerated dose (MTD) study of TGP18 was performed in female BALB/c mice. Single dose of TGP18 was determined and repeat dosing was performed twice a week for two weeks at different concentrations of TGP18 (0.5, 1, 2.5, 10 and 25 mg/kg) administered intravenously (IV). For therapy studies, female NCr nude mice (1-2 months old, weighing 18-20 g) were injected subcutaneously with 5×10^6^ cells (MDA-MB-231) or 1×10^6^ cells (A549) cells in the right flank region (1x HBSS (Hank's Balanced Salts Solution) + Matrigel). When the tumors were established (approx. 14 days, mean size 100-200 mm^3^), the mice were divided into three therapeutic groups with six mice/group for each xenograft model. The standard-of-care (SOC) drugs gemcitabine and doxorubicin hydrochloride were used as reference control for A549 and MDA-MD-231 xenograft model, respectively. TGP18 samples were dissolved in 5% DMA + 95% saline (Vehicle) to the required concentration and gemcitabine/doxorubicin directly dissolved in saline for IV administration. Vehicle control group animals were administered with vehicle alone. Tumor size was measured weekly twice using a digital Vernier calipers. Tumor volume was calculated as follows:

Tumor Volume = [Length (L) × Width (W)^2^] /2

The mice were weighed and examined at the same time to determine any signs of toxicity from the drug. Tumor growth inhibition (TGI) was calculated based on the following formula:


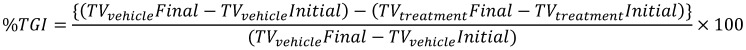


Group 1: Six mice were treated with a twice weekly dose of 0.5 mg/kg of TGP18 in 5% DMA+95% saline;

Group 2: Six mice were treated with a twice weekly dose of 100 mg/kg of gemcitabine (A549 xenograft model) or once weekly dose of 10 mg/kg of doxorubicin (MDA-MB-231 xenograft model) in saline;

Group 3: Six control mice were treated with 5% DMA+95% saline (vehicle) only, twice weekly.

Tumor weights were collected *ex vivo* after the mice were sacrificed following the completion of treatment period of 14 days. Mice were culled if tumors ulcerated or if a weight loss of 10-20% of the initial body weight was observed. We limited our study till two weeks due to the consequences of the tumors in some animals reaching the maximum permitted size.

### Immunohistochemistry

Immunohistochemical analysis of BCL-2 was performed as described previously 52. Antibody staining was performed on histological sections of formalin-fixed lung tumor xenografts. Briefly, the slides were re-hydrated in PBS for 15 min and the endogenous peroxidase was inhibited by 0.3% H_2_O_2_/methanol for 10 min at room temperature. For blocking, 5% skimmed milk/PBS was used for 30 min at room temperature. Slides were incubated with monoclonal anti-human Bcl-2 antibody (Abcam, ab196495) at 1:100 dilution for 16 h at 4 °C. The peroxidase-conjugated secondary antibody (Abcam) was used at 1:200 dilution and incubated for 1 h at room temperature. Staining was developed by immersing slides in 0.06% 3,3′-diaminobenzidine tetrahydrochloride (DAB) followed by counterstaining with Hematoxylin V.

### Tumor imaging studies

Tumor samples collected after sacrificing the mice were fixed in neutral buffered formalin solution (NBF). Tumors from mice M5 of group 2 (treated with 0.5 mg/kg of TGP18) and mouse M1 from group 1 (control) were snap-frozen and cut in 20 µm sections. These tissue sections were incubated with DAPI for 10 min and mounted on a glass slide. Images were acquired using a confocal fluorescence microscope (Olympus FV3000).

### Statistics

Statistical tests were performed with Prism (GraphPad). A value of *P* < 0.05 was considered statistically significant. All error bars represent standard deviation (S.D.). For quantitative data, statistical parameters are reported in the figure legends.

## Results

### Synthesis of neocuproine conjugates and their interactions with GQs

**Figure 1:**
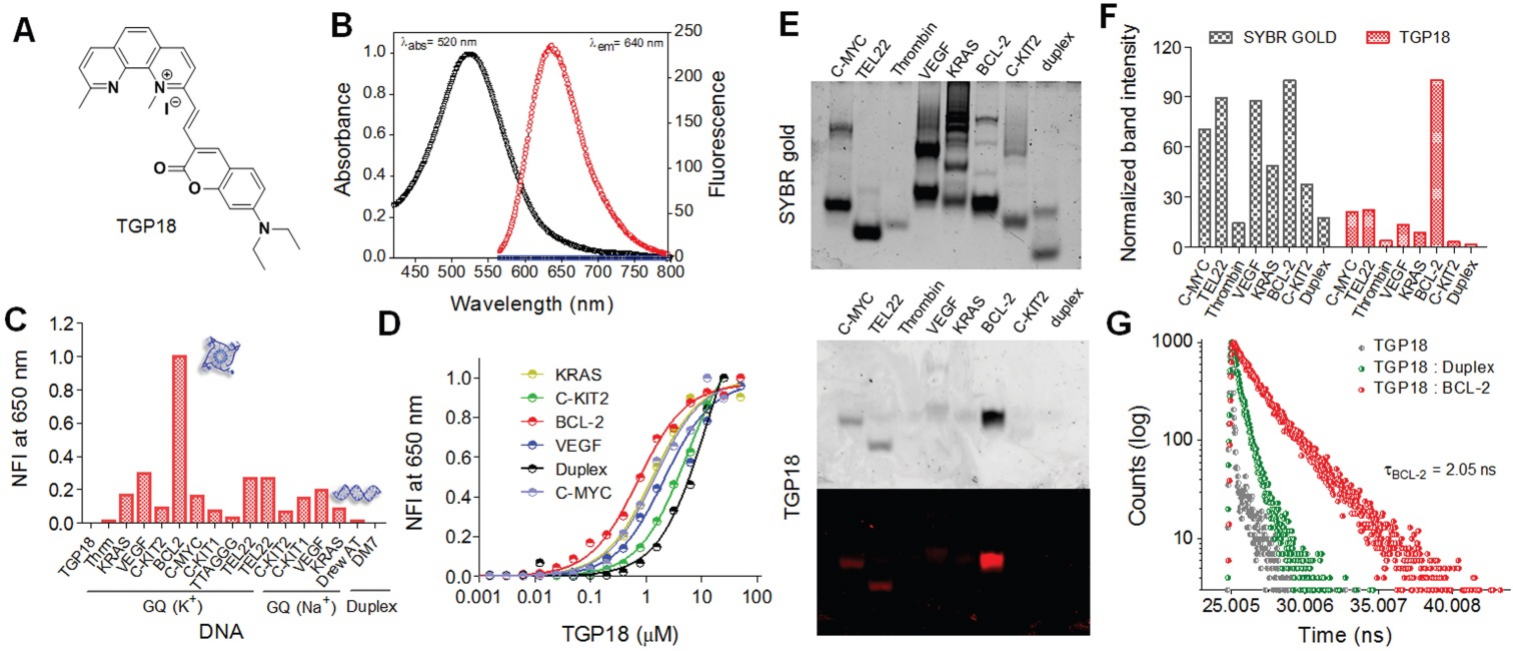
** Selective recognition and detection of BCL-2 GQ by TGP18 with turn on fluorescence response.** (**A**) Chemical structure of neocuproine conjugate TGP18. (**B**) Absorbance and fluorescence spectra of TGP18 in phosphate buffer (20 mM PBS, pH 7.4). (**C**) Normalized fluorescence intensity (NFI) of TGP18 in the presence of parallel or antiparallel GQ, and duplex DNAs. (**D**) The plot of NFI (F/F_o_) as a function of TGP18 concentration (0-30 µM) in presence of fixed concentrations of GQ and duplex DNAs. F_o_ and F represent the fluorescence intensity of TGP18 in the absence and presence of DNA, respectively. (**E**) Non-denaturing PAGE images of GQ and duplex DNAs stained with SYBR gold (top) and TGP18 (down), All DNA samples (2 μM each) were prepared in phosphate buffer (20 mM PBS, 100 mM KCl, pH 7.4). (**F**) Histogram of gel stained by SYBR gold and TGP18 using Image J software. (G) Fluorescence lifetime measurements of TGP18 in presence of BCL-2 GQ and duplex DNA.

The donor-acceptor neocuproine conjugates with extended π-conjugation were designed and synthesized by condensing the electron-deficient neocuproine with electron donor moieties such as coumarin and hydroxy julolidine carboxaldehydes (Figure 1A and Scheme S2). Knoevenegal condensation of neocuproine (2,9-dimethyl-1,10-phenanthroline) and methylated neocuproine (1,2,9-trimethyl-1,10-phenanthrolin-1-ium) with coumarin carboxaldehyde afforded compounds TGP17 and TGP18, respectively, while the condensation of methylated neocuproine with julolidine carboxaldehyde yielded TGP21 (Scheme S1-S2 and Figure S1-S7). The electron-donating group was varied to fine-tune the fluorescence properties of neocuproine derivatives. The photophysical evaluation revealed that TGP18 and TGP21 showed negligible fluorescence in buffer (20 mM PBS, pH 7.4), while TGP17 showed notable fluorescence (Figure S8). All conjugates were then subjected to preliminary fluorescence screening for *in vitro* interaction and selectivity towards various GQs and duplex DNA (see Table S1 for oligosequences). TGP21 showed weak emission at 660 nm (λ_ex_ = 575 nm), albeit with no change in fluorescence response in the presence of either GQ or duplex DNA (Figure S8C). Interestingly, TGP18 showed turn on fluorescence at 640 nm (λ_ex_ = 560 nm) for GQ and basal level fluorescence in the unbound state or in the presence of duplex DNA (Figure 1B). TGP17, a neutral analogue of TGP18 showed a moderate increase in fluorescence (λ_ex_ = 460 nm, λ_em_ = 540 nm) in presence of all GQs and duplex DNAs without any topology selectivity (Figure S8A). Moreover, the intrinsic fluorescence of TGP17 in buffer alone undermines its *in vitro* and *in cellulo* applications. The turn on fluorescence response of TGP18 towards GQs was further evaluated to assess the selectivity towards different GQ topologies under *in vitro* conditions employing various biophysical techniques.

### Specific interaction with BCL-2 GQ

We studied the binding interaction of TGP18 against 14 GQs of various promoter genes with distinct topologies (parallel, mixed, and anti-parallel topologies) and two duplex DNAs by monitoring the fluorescence response. As shown in Figure 1C, TGP18 (1 µM) exhibited significant fluorescence enhancement in the presence of BCL-2 GQ compared to other GQ topologies and duplex DNA. Remarkably, a 200-fold increase in fluorescence response around 640 nm accompanied by 10 nm blue shift was observed in the presence of BCL-2 GQ (Figure 1B and Figure S8B). In contrast, less than 70-fold response was observed for other GQs and almost negligible fluorescence for duplex DNAs. The dissociation constants (*K*_D_) calculated from the concentration-dependent fluorescence data showed a high affinity of TGP18 for BCL-2 GQ which is supplemented by 7-fold selectivity (Figure 1D). The determined *K*_D_ value revealed sub-micromolar affinity of TGP18 for BCL-2 (728±34 nM) over c-KIT2 (5.5±0.23 μM), C-MYC (1.5±0.19 μM), VEGF (2.12±0.35 μM), KRAS (1.3±0.13 μM) GQs and duplex (12.2±2.20 µM) DNAs. The selective turn on fluorescence and low *K*_D_ value for TGP18 for BCL-2 GQ complexation underline its high binding affinity compared to other GQ topologies. It is worth mentioning that TGP18 exhibited a strong red shift of 70 nm and maximum hyperchromism in the absorbance spectra upon titration with BCL-2 GQ (Figure S8B). The superior performance in terms of sub-micromolar affinity and enhanced fluorescence compared to other GQs and duplex DNAs unambiguously established the selectivity of TGP18 for mixed BCL-2 GQ structure. The data showed a linear relationship between fluorescence intensity of TGP18 and concentration of BCL-2 GQ in the range of 50-800 nM with a limit of detection (LOD) of 66 nM (Figure S9). To further validate the specific binding and fluorescence response of TGP18 to BCL-2 GQ, we performed gel electrophoresis and staining of various GQ and duplex DNA topologies. In the control experiment, SYBR gold, an extremely sensitive fluorescent dye for nucleic acids showed non-selective staining of all GQ and duplex DNA structures (Figure 1E). The quantified band intensities of TGP18 staining for various DNA structures are shown in Figure 1F, which established the specificity and potential utility of TGP18 for fluorescence identification and quantification of BCL-2 GQ *in vitro*. This data confirmed that TGP18 not only displays GQ selectivity over duplex DNA but preferentially discriminates BCL-2 GQ *in vitro* among other GQ containing promoter sequences with maximum turn on fluorescence response.

The selective and differential fluorescence response (steady-state intensity) alone is insufficient to assess the potential of a probe for cellular imaging of different DNA conformations owing to a possible difference in the cellular uptake and intracellular localization, which alter the effective concentration of the probe and in turn the intensity observed. On the other hand, fluorescence lifetime is concentration independent and therefore lifetime measurement of TGP18 in presence of GQs validate the steady-state fluorescence data and utility of the probe for *in cellulo* and *in vivo* imaging applications. In this context, time-correlated single photon counting (TCSPC) system was used to measure the fluorescence lifetime of TGP18 in the presence of different GQs (BCL-2, C-MYC, and TEL22) and a duplex DNA (Figure 1G and Figure S10). The lifetime traces were recorded in the presence of three-fold excess of DNA compared with the end point of steady-state emission titrations such that the concentration of the unbound probe is negligible. Interesting trends were observed in the lifetime measurements for different DNA topologies. The data revealed quenching of TGP18 fluorescence in solution (τ = 0.26 ns) in the absence of GQ, which is attributed to intramolecular rotation or torsional motion. In the presence of BCL-2 GQ, TGP18 showed 3-fold longer fluorescence decay time (τ = 2.05 ns) compared to duplex DNA (τ = 0.63 ns), which supports the observed steady-state intensity difference between GQ and duplex DNA (Figure 1G). In contrast, TGP18 showed shorter lifetimes of 1.5 ns and 1.6 ns in the presence of C-MYC and TEL22 GQs, respectively (Figure S10). The observed trend in fluorescence lifetime is found to be consistent with the steady-state fluorescence response and emission intensity of the probe in the presence of various GQs. The difference in fluorescence lifetimes among the GQs and duplex DNA conformations suggested that TGP18 is a potential optical probe to visualize GQs in live cells. The significant fluorescence enhancement and relatively longer fluorescence lifetime unambiguously established favorable recognition of BCL-2 GQ by TGP18 over other GQs and duplex DNA structures. The remarkable fluorescence turn on response of TGP18 in presence of GQ can be explained by the maximum π-electron overlap through molecular planarity attained in a constrained environment. The hydrophobic pocket created by the loop and groove of GQ provides a favorable constrained environment to restrict the intramolecular twisting of TGP18. To verify this premise, we measured fluorescence response of the probe titrating with increasing percentage of glycerol in phosphate buffer (Figure S11). TGP18 showed negligible fluorescence in low-viscosity buffer (0% glycerol) which gradually increased with percentage of added glycerol and exhibit strong fluorescence in high-viscosity buffer (80% glycerol). Addition of glycerol increases the viscosity of the buffer solution that mimics the constrained environment leading to restriction of free intramolecular rotation around the methine bridge separating coumarin and neocuproine moieties. In otherward, the constrained environment enforces TGP18 to attain planar molecular structure with maximum π-electron overlap required for strong turn on fluorescence response. Further, increase in glycerol percentage led to changes in the absorption spectra of TGP18 similar to that observed in presence of GQ. These results have established the origin of turn on fluorescence response of TGP18 in presence of GQ. These remarkable fluorescence properties motivated us to carry out detailed investigations on the specific recognition of BCL-2 GQ by TGP18.

### Hybrid binding interactions of TGP18 with BCL-2 GQ

Molecular dynamics (MD) simulations were undertaken to gain detailed insights on the mechanism of binding interaction of TGP18 with BCL-2 GQ at the atomic level. First, molecular docking studies were carried out, and molecular dynamics simulations and free energy calculations were performed for the selected least energy binding modes. In particular, the binding free energy was computed for the interaction of TGP18 with different oncogenic GQs (BCL-2, C-MYC, TEL22, C-KIT2, VEGF-A, and KRAS). The binding free energy values calculated using molecular mechanics-generalized Born surface area (MM-GBSA) approach identified TGP18 interaction with BCL-2 GQ (ΔG = -42.2 kcal/mol) as more energetically favored compared to other oncogenic GQs (ΔG < -25.0 kcal/mol). This high affinity binding of TGP18 leads to a sharp rise in the melting temperature (T_m_) of BCL-2 GQ by 8 °C compared to other GQ sequences (Figure S12). TGP18 showed groove interactions for all the parallel or antiparallel forming GQs (C-MYC, TEL22, C-KIT2, VEGF-A and KRAS) except for BCL-2 topology (Figure S13 and Table S3).

**Figure 2:**
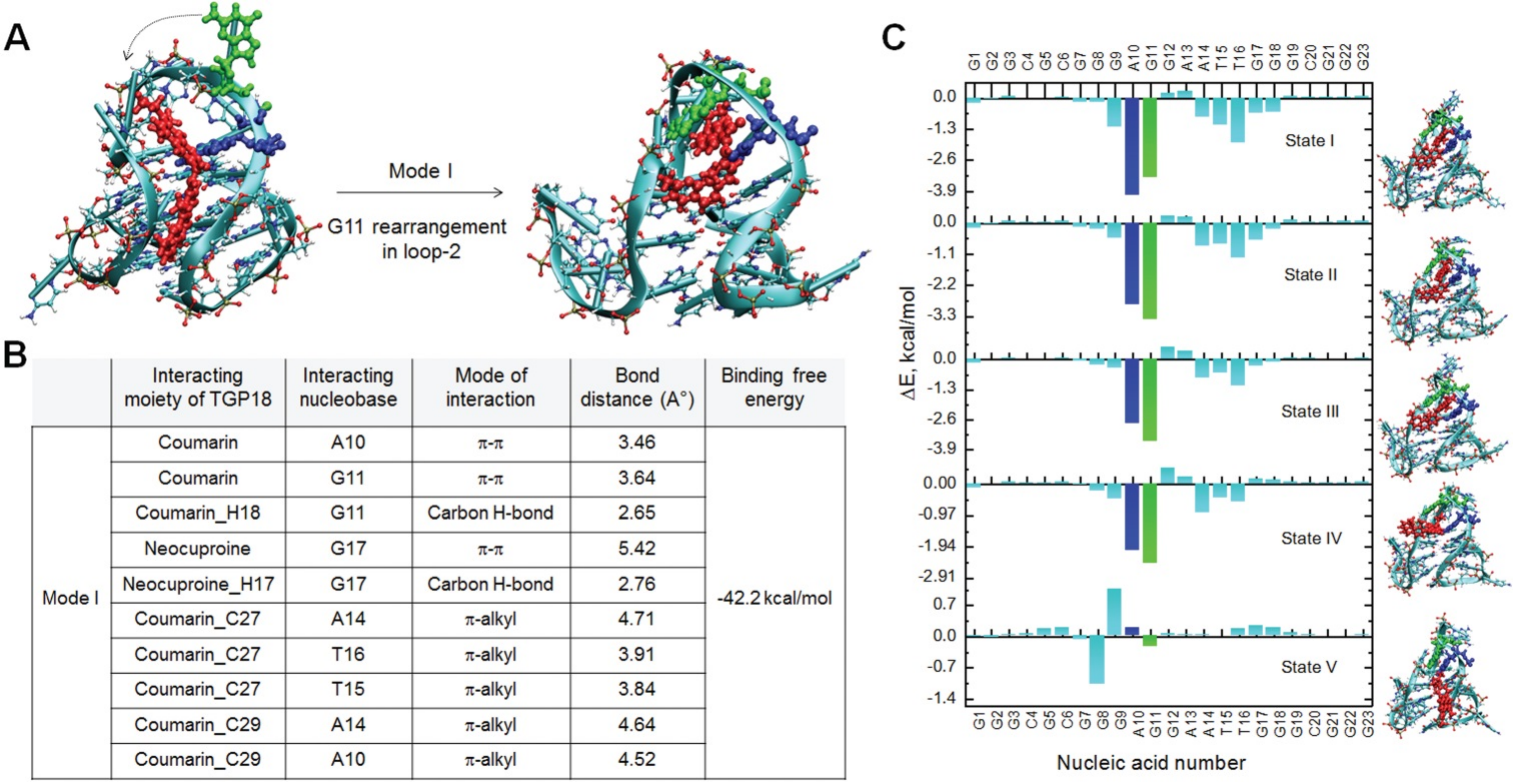
** Visualizations and molecular interactions of TGP18 with BCL-2 GQ in Mode I.** (**A**) Representative docked and simulated (50 ns long) configurations for BCL-2:TGP18 Mode I complex showing G11 (green) rearrangement to form π-stacking interactions with coumarin moiety of TGP18 (red) in loop-2. Mode I show hybrid binding involving coumarin moiety sandwiched between A10 (blue) and G11 (green) for loop-stacking and neocuproine moiety in groove interactions. (**B**) Summary of molecular interactions between TGP18 and BCL-2 in Mode I hybrid binding. (**C**) Nucleic acid wise contributions to the binding free energy along the egression pathway of TGP18 from BCL-2 GQ for the case of binding Mode I.

BCL-2 GQ forms a distinctive mixed hybrid (3+1) topology having two lateral loops (3 and 7-nucleotides respectively), a third single-nucleotide (nt) double-chain reversal loop (C) and four grooves (one wide, one narrow and two intermediate) of different widths 40. The second- long lateral loop conformation formed by 7-nt (A10-G11-G12-A13-A14-T15-T16) linker offered a unique binding space for TGP18. Nucleotides A10 and T15 present in this loop potentially form a base pairing (bp) through reversed Watson-Crick (WC) hydrogen bonding interactions and this bp stacks perfectly with 5'-end tetrad. Molecular docking studies yielded six different binding modes for TGP18-BCL-2 binding, for which MD simulations (total time scale 50 ns in each case) and binding free energies were computed (Figure S14 and S15). The six different binding modes are shown in Figure S14 and the binding free energies are tabulated in Table S4. Interestingly, TGP18 exhibited π-stacking interactions with A10:T15 bp present in loop-2 that contributed immensely for the two least free energy binding modes (ΔG_Mode I_ = -42.2 kcal/mol and ΔG_Mode II_ = -35.0 kcal/mol). In the most stable binding Mode I, coumarin and neocuproine moieties of TGP18 are involved in loop-stacking and groove binding interactions, respectively, whereas in mode II, coumarin and neocuproine moieties exchanged their roles for same interactions (Figure 2 and Figure S16). This hybrid mode of binding involving groove binding adjoined with loop-stacking interactions contributes immensely to the high affinity and selectivity of TGP18 for BCL-2 GQ. However, remaining low affinity binding modes (Mode III-VI) showed dominant π-stacking character without participating in binding to any groove regions. Further, in Mode I, G11 nucleotide present in the loop-2 that was exposed to solvent rearranged itself to come into spatial proximity of coumarin moiety which increased the π-stacking interactions (Figure 2B). Thus, coumarin moiety of TGP18 sandwiched between A10 and G11 nucleotides render hydrophobic contribution in the loop-2. Besides, the cationic neocuproine moiety of TGP18 was found to be involved in a multitude of electrostatic and weaker CH_3_-π interactions to impart the experimentally observed selectivity for BCL-2 structure (Figure 2B). There was no significant alteration in the conformation of G11 nucleotide of loop-2 in Mode II, which validated its lower affinity compared to Mode I (Figure S16).

The random acceleration molecular dynamics (RAMD) studies were carried out to further substantiate the conformational changes in loop-2 nucleotides, during unbinding pathways of TGP18 from BCL-2 structure (Figure 2C) 53. Five different configurations (state I-V) for Mode I binding along the egression (unbinding) pathway were collected and change in nucleotide residue wise contributions to binding free energy were estimated using MM-GBSA calculations and decomposition analysis (Figure S17 and Table S5). The change in nucleotide residue wise contribution was maximum for A10 (ΔE_A10_ = -4.0 kcal/mol) and G11 (ΔE_G11_ = -3.3 kcal/mol) compared to remaining nucleotides (ΔE ≥ -1.8 kcal/mol) of BCL-2 along the unbinding pathway for the binding Mode I as observed from the change in binding free energies. Along the egression pathway, binding free energies corresponding to A10 and G11 nucleotides varied drastically from state I (ΔE_A10_ = -4.0 kcal/mol; ΔE_G11_ = -3.3 kcal/mol) to state V (ΔE_A10_ = 0.18 kcal/mol; ΔE_G11_ = -0.23 kcal/mol) signifying their major contribution. The observed π-stacking contributions of A10 and G11 nucleotides for unbinding of TGP18 were considerably high, that corroborated with the G11 rearrangement shown in Figure 2A. In agreement with the binding free energies, the strong hydrophobic interactions offered by A10 and G11 nucleotides in binding Mode I resulted in the larger time scale for unbinding to reach the dissociated state when compared to other binding modes. This compelling evidence further confirmed that TGP18 is engaged in hybrid binding (loop-stacking and groove binding interactions) mode with BCL-2 GQ.

Next, we sought to understand the observed differential selectivity of three neocuproine conjugates (TGP17, TGP18, and TGP21) towards BCL-2 GQ over other GQs based on their relative binding free energies. The probes TGP17 and TGP21 show either loop-stacking or groove binding as the preferred binding sites (Figure S18) and the free energy data are given in Table S6. Interestingly, the binding mode of both the probes towards BCL-2 GQ showed loop-stacking character through hydrophobic π-stacking interactions and the next high affinity binding as the interaction with the minor groove of GQ. It is worth noting that although TGP17 displayed stronger van der Waals interactions with GQ, due to unfavorable electrostatic contributions (sum of electrostatic interaction with GQ and polar solvation energy which amounts to +40 kcal/mol) the binding free energy is increased when compared to TGP18. On the contrary, the penalty due to unfavorable electrostatic interactions is only +25 kcal/mol in the case of TGP18 and its binding to BCL-2 GQ is driven predominantly by van der Waals interaction (which amounts to -60 kcal/mol). The binding energies of neocuproine conjugates to BCL-2 GQ were found to be in the following order TGP18>TGP21>TGP17. Overall, these results validated the observed differential selectivity of neocuproine conjugates to GQs attributed to their donor-acceptor conjugation and cationic nature. These results substantiated the potential of hybrid binding mode comprising loop-stacking and groove interactions in achieving the subtle balance between binding affinity and selectivity for specific recognition towards BCL-2 GQ with sub-micromolar affinity.

### Therapeutic efficacy of TGP18

**Figure 3:**
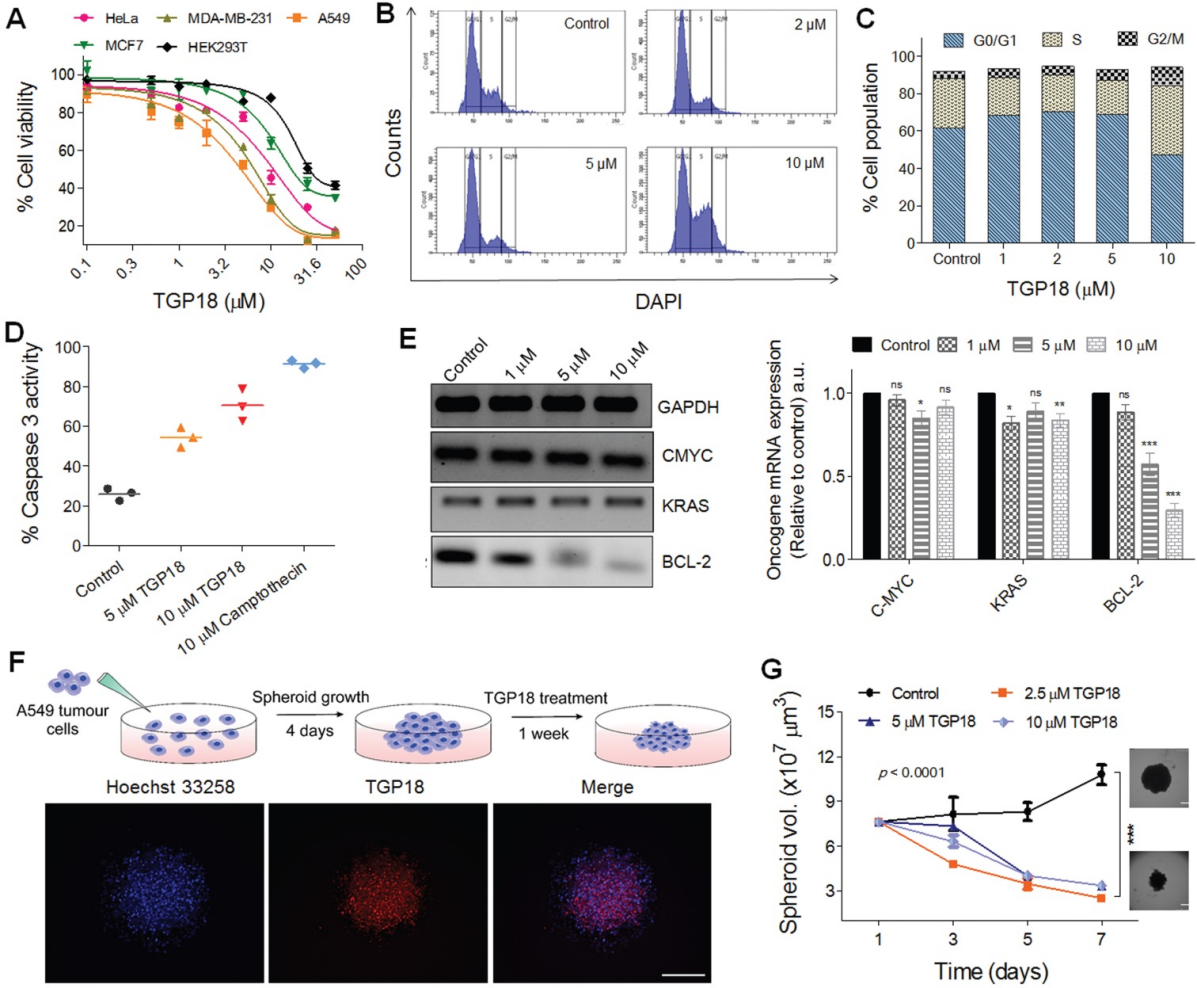
***In vitro* antiproliferative effect of TGP18 through apoptotic pathway.** (**A**) Antiproliferative effect of TGP18 on different cancer (HeLa, MDA-MB-231, A549, and MCF-7) and non-tumorogenic (HEK293T) cell lines treated for 24 h. (**B**) Cell cycle histogram of A549 cells after 24 h treatment with TGP18 at 2, 5 and 10 μM. Cells were analyzed by fluorescence-activated cell sorting (FACS) with the intensity of DAPI recorded. (**C**) The representative FACS profile shows the mean percentage of the cell population in G0/G1, S and G2/M phase (with 95% CIs) under different concentrations (1, 2, 5 and 10 μM) of TGP18; confidence intervals (CIs). (**D**) Effect of TGP18 (5 and 10 μM) and camptothecin (10 μM) treatment on caspase-3 activity in A549 cells for 24 h. (**E**) Transcription expression profile of C-MYC, KRAS, and BCL-2 upon treatment of TGP18 with increasing concentrations. Quantification of mRNA expression level relative to control (GAPDH) by RT-PCR in A549 cells. Error bars represent mean ± SE (N = 3). Statistical differences are determined compared to the control by two-tailed student's *t*-test (*P < 0.05, **P < 0.01, ***P < 0.001). (**F**) Fluorescence imaging of 3D tumor spheroid stained with TGP18 (500 nM, red) and Hoechst (blue). (**G**) Inhibition of 3D tumor spheroid (A549 cells) growth upon TGP18 treatment for 24 h. P values were determined by two-way ANOVA. scale bar, 100 μm.

Fluorescence probes selectively targeting GQs could be potential anti-cancer agents and are rarely investigated for its therapeutic efficacy *in cellulo*. We examined the antiproliferative effect of TGP18 in various cancer and non-cancer cell lines to establish the toxic and non-toxic concentration levels for therapeutic and diagnostic roles, respectively. Figure 3A illustrates the anti-proliferation effects of TGP18, as studied by MTT assay in cancer cell lines (HeLa, A549, MCF-7, and MDA-MB-231) and non-cancerous cell line (HEK293T) following treatment with the probe for 24 h. TGP18 showed relatively higher anti-proliferation effect in A549 and MDA-MB-231 cancer cells with IC_50_ (half-maximum inhibitory concentration) of 4 and 6 µM respectively, compared to HeLa and MCF7 (IC_50_ = 10-20 µM), while minimal cytotoxic effect was observed on normal HEK293T cells (IC_50_ > 25 µM). Among the cancer cell lines, TGP18 was found to be more effective in inhibiting the proliferation of A549 (lung cancer) and MDA-MB-231 (breast cancer) with low IC_50_ values compared to other cell lines. To understand the antiproliferative effect of the probe, we explored whether TGP18 can induce cell cycle arrest and apoptosis in A549 cells. Flow cytometry analysis was performed using 4′,6-diamidino-2-phenylindole (DAPI) staining to determine whether TGP18 mediated antiproliferation is associated with cell cycle arrest (Figure 3B). Treatment of cells with TGP18 (1, 2, 5 and 10 μM) significantly changed the cell cycle distribution compared to the untreated group. As the concentration of TGP18 is increased from 1 to 5 μM, cell cycle histogram showed an accumulation of G0/G1 population (61.6% to 70%) accompanied by S-phase decrease (26.5 to 18.3%) indicating the onset of apoptosis in a dose dependent manner (Figure 3C). At higher concentration, TGP18 showed a notable increase in the percentage of S-phase population from 26.5% (untreated cells) to 37.1% (10 μM), while a moderate increase in G2/M population from 4% (untreated cells) to 10% (10 μM) observed which indicates the arrest in S and G2/M-phase. A marked increase in the S-phase population indicates the arrest of cell cycle that mediates apoptosis induction. The observed S-phase arrest could be interfered with DNA damage as discussed in the later section.

Caspases play a key role in many forms of apoptosis and are critical constituents to catalyze the proteolytic cleavage of many key cellular proteins 54. We monitored the activation of caspase-3 in A549 cells to validate the apoptosis induction by TGP18 (Figure 3D). A549 cells were exposed to 5 and 10 μM TGP18 for 24 h and subjected to a fluorimetric caspase-3 activity assay. The activated caspase-3 cleaves the fluorogenic substrate to generate a fluorescence signal proportional to the number of apoptotic cells in the sample. The fluorimetric data showed dose dependent increase in the caspase-3 activation in treated cells and the activity was comparable to that of anti-cancer drug camptothecin (Figure 3D). Camptothecin treatment resulted in a marked induction of caspase-3 activity as shown by the increase in fluorescence. The increase in fluorescence response by TGP18 treatment was normalized to camptothecin treatment which is indicative of the relative increase in caspase-3 activity. Untreated cells did not show appreciable fluorescence due to negligible caspase-3 activity indicating no cellular apoptosis. Overall, the enhanced caspase-3 activity in treated A549 cells validated the apoptosis triggered by TGP18.

The anti-proliferative effect motivated us to evaluate the effect of TGP18 on transcriptional regulation of different oncogenes bearing GQs in A549 cells. Elevated expression of BCL-2 gene that encodes the anti-apoptotic BCL-2 protein greatly contributes to acquiring resistance for apoptosis in cancer cells, and its inhibition sensitizes cancer cells to apoptotic death 55. The qRT-PCR analysis in Figure 3E shows significantly downregulated BCL-2 expression at the transcription level with minimal effect on other oncogenes. TGP18 after 24 h treatment reduced the mRNA level of BCL-2 by ~70% in comparison to control. This data revealed that TGP18 upon GQ stabilization inhibits expression of BCL-2 at a cellular level, which corroborates with the observed *in vitro* GQ binding affinity. As the mounting evidence indicates that the cell cycle and apoptosis are inextricably linked, BCL-2 downregulation in combination with cell cycle arrest supports the notion of apoptosis induced by TGP18 accounting for its anti-proliferative effects.

### *In vitro* tumor spheroid inhibition

Spheroids produced by the 3D culture techniques play a crucial role in the study of tissue biology and serve as a powerful tool for the investigation of cancer progression. *In vitro* anti-cancer efficacy of TGP18 studied in 2D monolayer cells was further validated in the *in vivo* tumor mimicking 3D spheroids formed by A549 cancer cells. 3D spheroids were prepared by following reported procedure as shown in Figure 3F 56. The inherent far-red fluorescence response of TGP18 allowed us to monitor the tumor penetration ability in 3D spheroids. The 3D spheroids of A549 cells were treated with TGP18 and nuclei were stained with Hoechst (Figure 3F). Fluorescence microscopy images revealed uniform distribution of TGP18 both at the periphery and core of 3D spheroid. The efficient spheroid penetration ability of TGP18 encouraged us to study its effect on the 3D spheroid growth after the treatment. The growth delay experiment was performed, and volume of the spheroid was quantified for up to 7 days, from the day of the treatment 56. Significant inhibition of growth was observed in the case of TGP18 (2.5, 5 and 10 µM) treated spheroids, while the spheroid volume increased substantially in untreated control group (Figure 3G). This *in vivo* mimicking tumor spheroid inhibition thus provided a valuable impetus to study the anticancer activity of TGP18 in the animal model. Initially, the *ex vivo* erythrocyte (red blood cell) hemolysis assay was performed to screen for toxic hemolysis prior to *in vivo* study wherein different concentrations (0.1 to 100 µM) of TGP18 was co-incubated with collected human red blood cells in PBS (Figure S19). The amount of hemoglobin released during the incubation period was quantified as a measure of red blood cell lysis, which is normalized to the amount of hemoglobin released in positive control samples lysed with a detergent. The therapeutic concentration window of TGP18 (5-20 µM) showed negligible hemolysis (< 10%), while higher concentrations induced hemolysis gradually with time. Therefore, the minimum hemolysis associated with TGP18 suggests better suitability and biocompatibility for therapeutic application.

### TGP18 induce nucleolar stress, DNA damage and oxidative stress response

**Figure 4:**
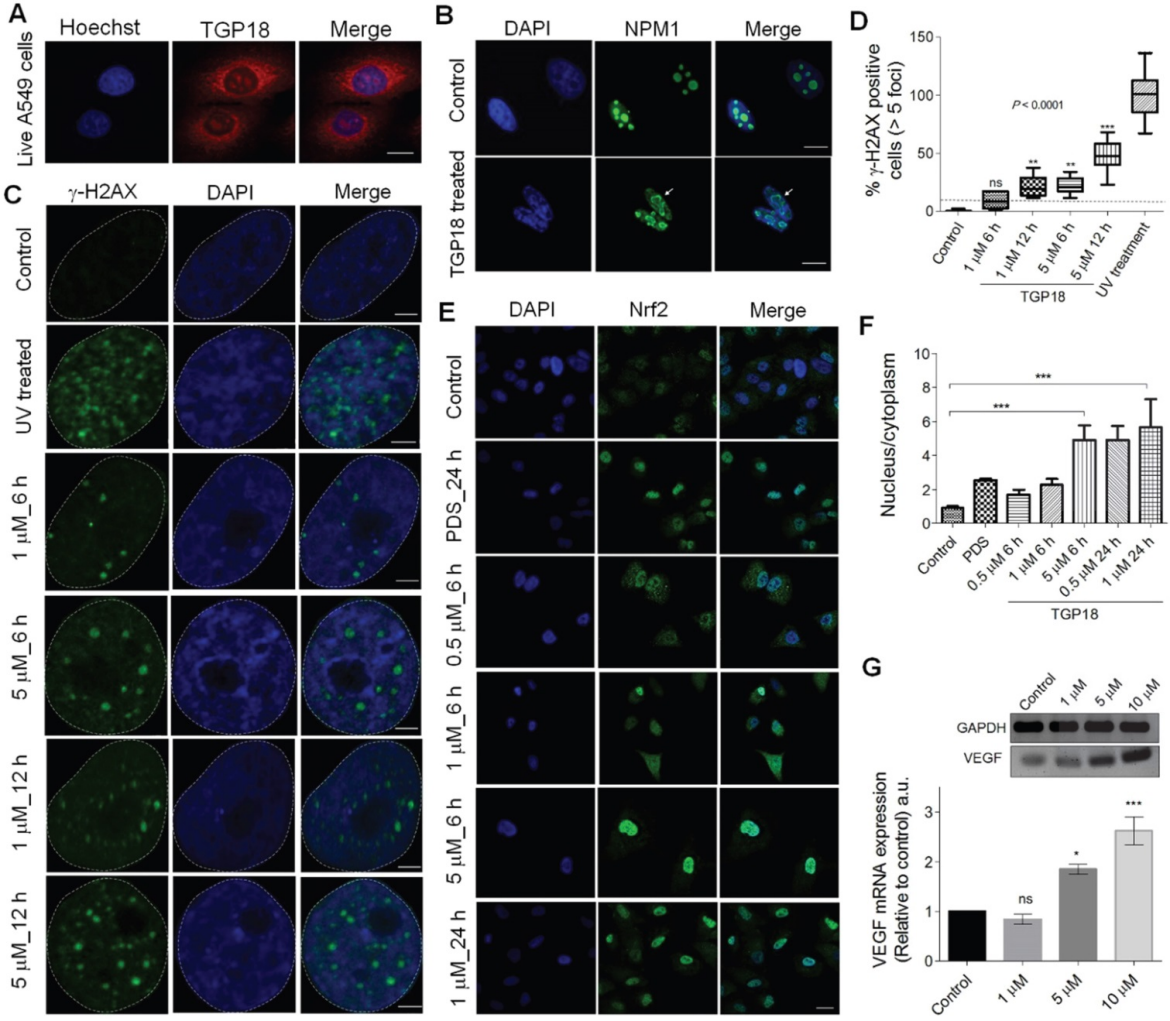
** Cellular localization in A549 cells, and induction of DNA damage and oxidative stress by TGP18.** (**A**) Confocal images of A549 cells showing cellular uptake of TGP18 (300 nM) incubated for 1 h. The cells were co-stained with nuclear staining dye Hoechst 33258. Scale bar, 25 μm. (**B**) Representative immunofluorescence images of NPM1 (green) in A549 cells treated with 1 µM TGP18 for 0.5 h. Arrow indicates nucleolus distortion in A549 cells. Cell nuclei were stained with either DAPI (blue). Scale bar, 10 μm. (**C**) DNA damage response (DDR) as a result of treatment with TGP18 or UV exposure. Cells were treated with TGP18 (1 and 5 μM) for 6 and 12 h or exposed to UV for 1 h. The formation of γ-H2AX foci (green) was detected by the immunofluorescence experiments. The nuclei were stained with DAPI (blue); Scale bar, 2 μm. (**D**) Quantification of γ-H2AX positive cells. Asterisks indicate statistical significance determined by the Kruskal-Wallis nonparametric test of treated in comparison with untreated cells. (**E**) Oxidative stress response (OSR), upon treatment with TGP18 or PDS. Representative immunofluorescence images of A549 cells showing nuclear translocation of Nrf2 (green) upon treatment with TGP18 (0.5, 1 and 5 µM) or PDS (10 µM) for 6 and 24 h. The nuclei were stained with DAPI (blue); scale bar, 10 μm. (**F**) Nrf2 translocation quantified using nucleus to cytoplasm Nrf2 signal ratios. Asterisks indicate statistical significance determined by one-way ANOVA of treated in comparison with untreated (control) cells, ***P < 0.001. (**G**) Expression profile of VEGF transcription upon treatment of TGP18 with increasing concentrations. Quantification of the mRNA expression level of VEGF relative to control (GAPDH) by RT-PCR. Error bars represent mean ± SE (N = 3). Statistical differences are determined by one-way ANOVA of treated compared to the control by (*P < 0.05, ***P < 0.001).

Cellular uptake of TGP18 was investigated over a wide range of incubation times (0.5-2 h) at its non-toxic (to normal cells) concentration range (100-500 nM) in A549 cancer cell line. Fluorescence imaging of A549 cells illustrated the accumulation of TGP18 in the cytoplasm and nucleolus of both live and fixed cells (Figure 4A and Figure S20A). Adequate literature available to support the role of nucleolus in the regulation of cell cycle, stress response and apoptosis 57. We validated the nucleolus uptake of TGP18 in A549 cells using nucleolus protein marker nucleophosmin (NPM1). NPM1 is one of the most abundant non-ribosomal nucleolar proteins like nucleolin, play a key role in the ribosome biogenesis, and implicated in ribosome maturation and response to stress stimuli 57-59. The specific localization of TGP18 in the nucleolus likewise observed by many GQ targeting ligands, prompted us to investigate its effect on the nucleolar distribution through NPM1 12, 18, 60-64. TGP18 (1 µM) treatment indeed influenced NPM1 nuclear localization within A549 lung cancer cells. Data in Figure 4B shows that 0.5 h after the treatment with TGP18, NPM1 starts to redistribute outside the nucleolus which resulted in almost completely diffused nuclear staining compared to the untreated control cells (Figure S20B). Earlier reports have established a direct link between NPM1 nucleolar localization and its GQ-binding properties. NPM1 recognizes GQ structures found at rDNA genes both *in vitro* and *in vivo* 65. Therefore, we have shown here that GQ stabilizing ligand TGP18 displaces NPM1 from its native compartment that establishes the competitive effect of TGP18 on NPM1 binding with GQ. The translocation of NPM1 to nucleoplasm was also accompanied by the nucleolus distortion wherein nucleoli were no longer visible as phase-dense and compact, rather appeared distorted from the normal round-like shape. The alterations in the nucleolar structure and function induced by TGP18 promotes nucleolar stress that eventually plays role in the apoptotic pathway 59, 66. NPM1 re-distribution in the nucleoplasm and nucleolar stress further indicate high affinity of TGP18 towards GQ structure. Although, evidence for NPM1 role in multiple aspects of ribosome biogenesis is substantial, its redistribution and induced nucleolar stress in response to DNA damage response (DDR) have emerged recently 67. Moreover, an intriguing effect of GQ stabilizing ligand is to induce DNA damage and genome instability 60, 68.

Next, we sought to assess whether TGP18 induced nucleolar stress signals and cell cycle arrest at S-phase (Figure 3B) translate into a cellular response of DNA damage and oxidative stress. To evaluate the TGP18 triggered DNA damage response (DDR) in A549 cells, we have investigated the formation of DNA damage foci due to the presence of phosphorylated H2AX (γ-H2AX), a phosphorylated variant of histone 2A that associates with DNA double-strand breaks 69. Cells treated with TGP18 (1 and 5 µM) for 6 and 12 h were analyzed by immunofluorescence for γ-H2AX-containing foci (Figure 4C). A marked increase in DNA damage as a function of incubation time (6 and 12 h) and concentration (1 and 5 µM) was observed. In a control (positive) study, A549 cells were irradiated with UV radiation that caused DNA damage extensively [97.81±4.07 (N = 23), *P* < 0.0001] as seen from the high amounts of γ-H2AX foci (Figure 4C). TGP18 induced significant DNA damage [47.81±2.64 (N = 23)] at higher concentration (5 µM for 12 h) as compared to 5 µM at 6 h [20.54±1.56 (N = 23)] and lower concentration of 1 µM [8.96±1.33 (N = 23)] (Figure 4D). This data suggests that TGP18 at therapeutic concentration window induce pronounced DNA damage signaling upon GQ binding for the onset of cellular apoptosis. The DDR signaling induced by TGP18 correlates well with the observed cell cycle arrest in G2/M and S phase, and apoptosis 70, 71. DNA damage and nucleolar stress are possibly accompanied by synergistic cellular stress response which motivated us to assess oxidative stress in cells following TGP18 treatment.

**Figure 5:**
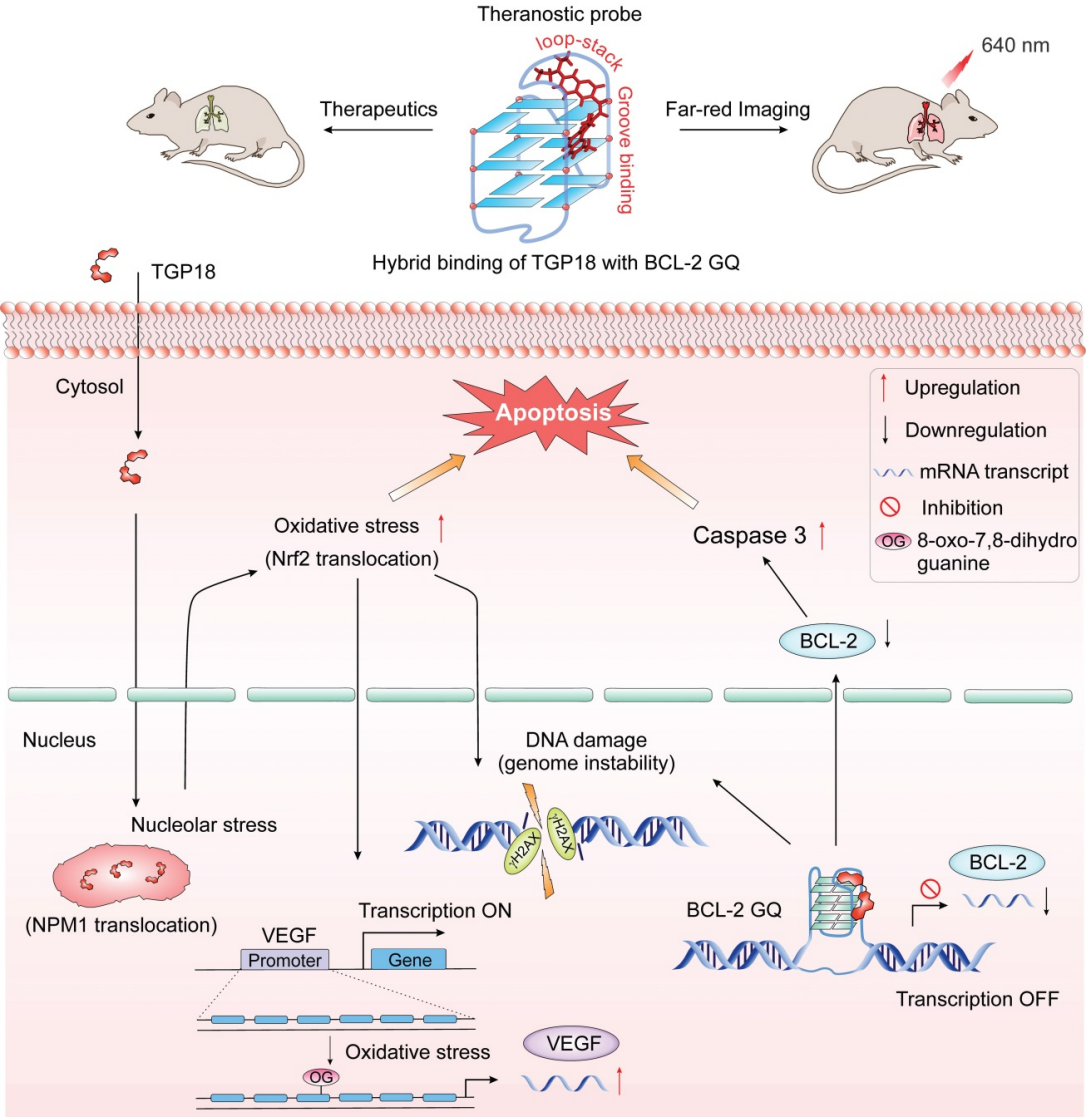
** Theranostic action by TGP18 in lung cancer.** Schematic representation shows quadruplex stabilization at BCL-2 promoter and nucleolar stress caused by TGP18 promotes apoptosis via signaling cascade of genome instability (DNA damage) and oxidative stress in A549 lung cancer cells. Top panel shows the theranostic (therapeutic efficacy and far-red imaging at 640 nm) activity of TGP18 in the lung cancer xenograft model upon BCL-2 stabilization through hybrid binding mode (loop-stacking and groove interaction).

The activation of oxidative stress response (OSR) upon GQ stabilization is seldom explored for any reported GQ ligands. Herein, activation of OSR stimulated by TGP18 was evaluated in A549 cells. Nuclear factor erythroid 2-related factor 2 (Nrf2), a transcription factor mediates the antioxidant response in association with the Kelch like ECH associated protein 1 (Keap1) 72, 73. Under unstressed conditions Keap1 keeps Nrf2 in the cytoplasm, while oxidative stress leads to oxidation of Keap1 causing its dissociation from Nrf2 and translocation of the later to the nucleus 74. Therefore, translocation of Nrf2 from cytoplasm to the nucleus is a reliable indicator to assess OSR 75. Cells treated with increasing concentrations of TGP18 (0.5, 1 and 5 µM) for 6 h were analyzed by immunofluorescence which showed significant accumulation of Nrf2 in the nucleus inferring its translocation (Figure 4E). The untreated cells (control) did not show a noticeable accumulation of Nrf2 in the nucleus revealing the quiescent condition. TGP18 showed time-dependent effect as revealed by the increased Nrf2 in the nucleus at 24 h compared to 6 h of incubation time (Figure 4E). The oxidative stress induction was found to be concentration dependent as revealed by the data wherein 5 µM of TGP18 showed much elevated levels of Nrf2 in the nucleus compared to 1 µM and 0.5 µM treatments. Pyridostatin, a GQ stabilizer, and DNA damage 60 inducer served as a positive control which induced Nrf2 translocation and accumulation in nucleolus under OSR similar to TGP18 (Figure 4F). Nevertheless, a high flux of reactive oxygen species (ROS) produced in response to oxidative stress shown to play an important signaling role by invoking the oxidative modification of promoter DNA sequences that entails transcription regulation 76-79. Cynthia Burrows and coworkers reinforced this notion by providing a mechanistic framework on which a canonical oxidative DNA damage product, 8-oxo-7,8-dihydroguanine (OG) occurs in the GQ forming promoter sequence of VEGF (vascular endothelial growth factor) gene that directly activates the transcription and up-regulates VEGF expression level 76, 80. Besides providing evidence of OG as an epigenetic modification, an increase in VEGF expression is considered a critical marker for monitoring oxidative stress. Thus, we assessed VEGF expression levels in TGP18 treated A549 cells. As shown in Figure 4G, cells treated with TGP18 for 24 h revealed upregulation of VEGF expression at the transcriptional level in a dose dependent manner (1, 5 and 10 µM), thus corroborating with the induced OSR. As a result, Nrf2 translocation and upregulated VEGF expression recapitulated the features of oxidative stress as a possible mutual pathway for DDR, cell cycle arrest and apoptosis induced by TGP18 (Figure 5).

### *In vivo* anticancer activity in breast and lung cancer model

**Figure 6:**
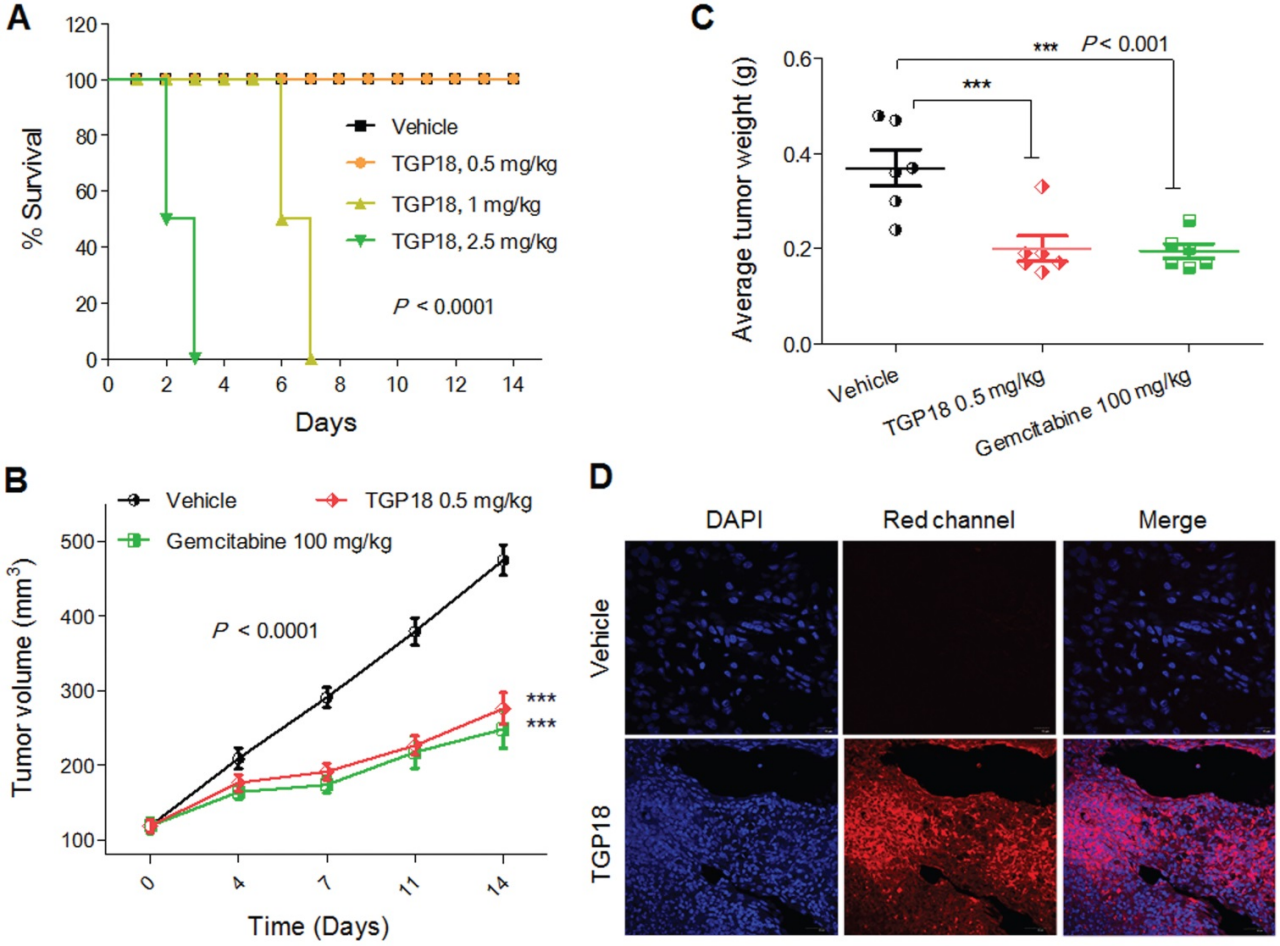
** GQ stabilization markedly suppressed the growth of A549 tumor xenografts.** (**A**) Kaplan-Meier analysis of survival for the mice in four experimental groups, receiving either TGP18 (0.5, 1 and 2.5 mg/kg) or vehicle (control). (**B**) Tumor growth suppression in mice (n = 6) treated with TGP18 and gemcitabine compared with vehicle treated mice. (**C**) Average tumor weight of mice (n = 6) for the three experimental groups monitored after 14 days of treatment. *P* values were determined by one-way ANOVA followed by Dunnett's test for treated compared to control (vehicle). (**D**) Images (x 40) of tissue sections close to the tumor surface of A549 xenografts treated with either vehicle or TGP18 followed by nuclear staining with DAPI. The fluorescence images were obtained in the blue channel (DAPI) and red channel (TGP18).

To validate the potential anticancer activity of GQ binding agent TGP18, we evaluated *in vivo* anti-tumor efficacy in mouse xenograft models of breast cancer (MDA-MB-231) and human lung carcinoma (A549) established in Athymic Nude mice (Figure 6 and Figure S21). The maximum tolerated dosage (MTD) for intravenous administration of TGP18 was found to be 0.5 mg/kg without any treatment-related adverse effects in terms of body weight and clinical toxicity signs (Figure 6A). A therapeutic schedule of two doses per week was followed with the MDA-MB-231 breast and A549 lung tumor xenograft models, and each dose of 0.5 mg/kg was administered over a period of two weeks. In the breast cancer (MDA-MB-231) model, TGP18 treatment at 0.5 mg/kg resulted in a marginal tumor growth inhibition of 43% (*P* < 0.001) compared to the vehicle treated control group (Figure S21). The reference compound doxorubicin showed 90% tumor growth inhibition at 10 mg/kg body weight, which was statistically significant (*P* < 0.0001). TGP18 showed moderate *in vivo* anti-tumor activity in breast cancer in contrast to the considerable anti-proliferation effect observed *in vitro*. Interestingly, TGP18 showed a dose-dependent anti-tumor response in lung cancer (A549) model with the minimal dose of 0.5 mg/kg to produce a significant growth inhibition (*P* < 0.0001) in comparison with 100 mg/kg dose of gemcitabine, a reference anticancer drug used in the study (Figure 6B). At 14 days of the therapeutic dosage experiment, an average of ca., 56% and 64% reduction in tumor growth was observed for TGP18 and gemcitabine treated mice, respectively, which was statistically significant compared to vehicle treated group and studies were performed over the group of six animals (Figure 6B). Remarkably, 0.5 mg/kg dosage of TGP18 showed anti-tumor efficacy comparable to that of 100 mg/kg dose of gemcitabine which is 200-fold higher than the former. For the entire duration of the study, no significant changes in the body weights or adverse effects such as tumor ulceration were observed for TGP18/gemcitabine treated mice (Figure S22). The *ex vivo* tumor weight of different treatment groups collected after completion of 14 days treatment showed an average 50% reduction in weight compared to the control group, which further confirmed the significant tumor growth inhibition by TGP18 (Figure 6C). We found that TGP18 drastically slowed the growth of A549 flank tumors and significantly prolonged the survival in lung tumor bearing A549 xenograft mouse (Figure S23). The *in vivo* anti-tumor activity of TGP18 shown to be more effective in lung cancer (A549) model with significant growth inhibition compared to breast cancer (MDA-MB-231) model. Thus, *in vivo* results confirmed the admirable tumor-suppressive efficacy of TGP18 at a 200-fold lower dosage compared to a reference drug (gemcitabine). In addition, immunohistochemical staining of BCL-2 gene was performed on formalin fixed paraffin embedded tumor sections (Figure S24). The distribution of BCL-2 expression was mostly observed in the cytoplasm and shown to be significantly higher in the vehicle control treated tumor sections. However, immunostaining was weakly present in sections treated with TGP18 or gemcitabine, demonstrating the reduction of the target gene BCL-2. This study further corroborates with the *in cellulo* studies verifying the mechanistic action of TGP18.

Further, the intrinsic turn on fluorescence of TGP18 with emission wavelength in the far-red region (640 nm) has readily enabled the monitoring of cellular and tissue uptake. The tumor sections collected close to the tumor surface were imaged at far-red wavelength using confocal microscopy at different depths to visualize the distribution of TGP18 in A549 tumor tissue samples (Figure 6D and Figure S25). Bright fluorescence detected from tissue sections indicates the theranostic activity of TGP18 at the tumor site by its deep penetration ability and good uptake. The turn on fluorescence intensity was approximately 9-fold that of control tissues. Thus, TGP18 meets the criteria of imaging and therapeutic activity, which verified and validated the rationale of our GQ-targeted theranostic probe design.

## Discussion

Theranostic agent with therapeutic and diagnostic capabilities can significantly aid the disease management and provide a means for creating more effective treatments for cancer. Regulation of GQ-mediated adverse cellular processes using designer chemical tools is a promising strategy to develop effective therapeutic interventions to combat cancer. Development of drug candidates based on small molecule fluorescence probes with selective targeting of oncogenic GQs offer effective diagnostic therapy for cancer with minimal off-target effects. However, small molecules with significant therapeutic efficacy coupled with appreciable imaging potential are rare. In this study, we designed neocuproine-based probe with the rationale of achieving specific recognition of GQ topology through hybrid binding and anti-cancer activity that offers an unprecedented scope to develop GQ-targeting theranostic agents. The π-conjugation of neocuproine acceptor-moiety with donor moiety was conceived in our probes design (TGP17, TGP18 and TGP21) to achieve maximum π-electron overlap through molecular planarity in a constrained environment offered by the loops and grooves of GQ topology.

Despite the minimal difference in the molecular structure of neocuproine conjugates, TGP18 offered an exceptional turn on fluorescence response with BCL-2 GQ topology and anti-cancer properties in the xenograft model. The observed selectivity of the promising probe TGP18 is attributed to optimized donor-acceptor chemistry of constituent units viz., coumarin and neocuproine. The differential selectivity of TGP18 for BCL-2 GQ over various other GQs and duplex DNA was assessed by detailed steady-state and lifetime fluorescence studies, gel electrophoresis staining, supported by thermal melting analysis and computational modeling. These studies have supported the GQ selectivity over duplex DNA and, the topology selective-recognition of BCL-2 GQ by TGP18 with a sub-micromolar affinity (K_D_ = 0.73 µM), while the rest of the conjugates showed poor or moderate binding affinity towards GQs without any selectivity. The GQ binding specificity of TGP18 towards BCL-2 promoter is promising, as most of the highly potent GQ stabilizing ligands shown to have promiscuous binding to all GQs with little or no discrimination which is probably attributed to their low nanomolar K_D_ values 81.

Most of the ligands with aromatic π-conjugation are developed to bind GQs via external π-π stacking interactions 82. These ligands with mere π-π stacking interactions failed to show specific target recognition among the myriad of GQs resulting in unfavorable pharmacokinetics due to off-target effects. For the first time, we recognized loops as the major conformational elements that define structural variability in GQs and not the common G-quartet structure. The length and folding pattern of loops result in variable and flexible cavities that offer specific binding sites for suitably designed probes/drug candidates 83. For instance, BCL-2 GQ (PDB: 2F8U) is the only structure exhibiting a long loop (7 nt) and two shorter loops (3 and 1 nt) whereas C-MYC, C-KIT and TEL22 have loops ≤ 4 nt 40, 84-86. Moreover, the groove widths of BCL-2 GQ (10.3, 6.4, 17.5 and 7.7 Å) are distinctly different from C-MYC GQ (9.7, 14.9, 12.7, 11.9 Å) and others. The distinct loop conformation and resulting groove found within the BCL-2 GQ present differential interaction sites that perfectly suits the specific binding of TGP18 through a unique hybrid binding mode (loop-staking and groove binding). We correlated this differential binding affinity using *in silico* modeling of TGP18-GQ complexes in explicit solvent and subsequent binding free energy calculations. It was found that the binding affinity and selectivity of TGP18 was driven by van der Waals interactions assisted hybrid binding mode involving both loop-stacking and groove binding with BCL-2 GQ. Interestingly, the coumarin moiety of TGP18 induced conformational rearrangement of G11 base present in the in the flexible 7 nt lateral loop-2 for facilitating strong π-stacking interactions while the neocuproine core stayed in the groove of GQ. Groove binding of neocuproine moiety offered additional π-π and π-alkyl interactions besides coulombic interactions driven by its intrinsic cationic charge (with negatively charged phosphate groups) which offered a bona fide advantage to maintain the integrity of the hybrid binding interaction. Although electrostatic interactions are dominant, they are nullified by the polar solvation energy contributions making the van der Waals interaction (refer to Table S5) as the sole driving force behind the selective recognition of BCL-2 GQ over other GQ structures. These results highlight the potential of hybrid binding mode involving loop-stacking and groove interactions in achieving the favorable binding affinity and selectivity for turn on fluorescence recognition of BCL-2 GQ.

The detailed *in cellulo* investigations perpetuate the role of TGP18 in the downregulation of BCL-2 transcription to induce apoptosis bestowing anti-tumor efficacy towards lung cancer cell line (A549). Accumulating evidence suggests that replicative stress is a common feature of lung cancer. In fact, therapeutic approaches like DNA damaging chemotherapies are known to exacerbate such stress and kill lung cancer by replicative damage 8, 87. Our experimental data showed that TGP18 contributed to replication stress as evidenced by the S-phase arrest, which further synergized with DDR as high levels of DNA damage shown by γ-H2AX foci, oxidative stress (Nrf2 translocation) and nucleolar stress (NPM1 translocation) to promote apoptosis in cancer cells. The recognized connection between DNA damage (genome instability) and replicative stress in sensitizing lung cancer cells to apoptosis, invigorates the preferential anti-cancer activity of TGP18 towards lung cancer 88. Besides the role of TGP18 in mediating cellular stress responses, VEGF expression was upregulated in response to oxidative stress demonstrating the crosstalk between cellular responses and gene expression. The production of oxidative stress by TGP18 was found to be dose dependent and correlated with the concentration dependent increase in DNA damage. Thus, the synergized signaling events of DDR, OSR and nucleolar stress provide mechanistic insights in linking the genome instability and replicative stress in response to GQ stabilization by TGP18 (Figure 5).

These mechanistic insights underscore the excellent therapeutic efficacy of TGP18 studied *in vitro* 3D tumor spheroid and *in vivo* A549 xenograft model via GQ stabilization. Remarkably, a lower dosage of TGP18 (0.5 mg/kg) showed anti-tumor activity on far with the reference drug gemcitabine (100 mg/kg). Moreover, the moderate activity of TGP18 in the breast cancer *in vivo* model reveals the preferred therapeutic action towards lung cancer. The reported association of lung cancer with increased levels of oxidative stress markers (e.g., OG) and decrease in DNA damage repair rate, partially attenuates the sensitivity of TGP18 towards lung cancer 89. However, further investigation may be necessary to fully understand the mechanism of action. The therapeutic agent TGP18 found to reach the target tumor site as monitored by its far-red imaging of the tumor tissue, which completes the theranostic procedure as per our design strategy.

## Conclusion

In conclusion, we described the theranostic activity of TGP18 by turn on fluorescence recognition of BCL-2 GQ through unique hybrid binding mode, its anti-lung cancer activity and tissue imaging potential. The neocuproine core together with coumarin moiety of TGP18 aided in achieving a fine balance between binding affinity and selectivity towards BCL-2 GQ through dual loop-groove interactions. Our strategy of specific topology recognition through hybrid binding mode led to capitalize on the gains of oxidative stress and genome instability to kill lung cancer cells *in vivo*. In addition, TGP18 with turn on emission band at the lower edge of far-red to NIR spectroscopic window proved to be a viable probe for tumor tissue imaging. Collectively, theranostic agent TGP18 with outstanding biocompatibility showed *in vivo* tumor inhibition and tissue imaging, indicating an excellent clinical translational potential. These results lay the foundation for uncovering the design of novel GQ-specific fluorescent ligands with combination of therapeutic and diagnostic potential for diagnostic therapy in diverse human cancers.

## Supplementary Material

Supplementary figures and tables.

## Abbreviations

GQ: G-quadruplex
BCL-2: B-cell CLL/lymphoma 2
DMEM: dulbecco's modified eagle medium
FBS: fetal bovine serum
DMSO: dimethyl sulfoxide
MTT: 3-(4,5-dimethylthiazol-2-yl)-2,5-diphenyltetrazoliumbromide
DAPI: 4′,6-diamidino-2-phenylindole
PDS: pyridostatin
PBS: potassium phosphate buffer
KCl: potassium chloride
NaCl: sodium chloride
DCM: dicholoromethane
MeOH: methanol
HRMS: high resolution mass spectra
HPLC: high performance liquid chromatography
TCSPC: time-correlated single photon counting
RAMD: random acceleration molecular dynamics
RT-PCR: real time polymerase chain reaction
FACS: fluorescence-activated cell sorting
DDR: DNA damage response
NPM1: nucleophosmin
Nrf2: Nuclear factor erythroid 2-related factor 2
OSR: oxidative stress response
SD: standard deviation
