# Supplementary material for: Recognition of G-quadruplex topology through hybrid binding with implications in cancer theranostics
Source: Theranostics. 2020 Aug 20;10(23):10394–414. doi: 10.7150/thno.48675 (PMC7482797; doi:10.7150/thno.48675)
Supplement: Supplementary file 1 — Supplementary figures and tables. [file thnov10p10394s1.pdf]

Supplementary Material

Recognition of G-quadruplex topology through hybrid binding with implications in cancer theranostics

Yelisetty Venkata Suseela,[1] Pardhasaradhi Satha,[1] N. Arul Murugan,[2] and Thimmaiah Govindaraju[1*]

[1]Bioorganic Chemistry Laboratory, New Chemistry Unit, Jawaharlal Nehru Centre for Advanced Scientific Research, Jakkur P.O., Bengaluru 560064, Karnataka, India

[2]Department of Theoretical Chemistry and Biology, KTH Royal Institute of Technology, S-106 91, Stockholm, Sweden

* Corresponding author:

Thimmaiah Govindaraju, Ph.D
Associate Professor
Bioorganic Chemistry Laboratory
New Chemistry Unit
Jawaharlal Nehru Centre for
Advanced Scientific Research (JNCASR)
Jakkur P.O., Bengaluru-560064, India
Tel: +91 80 2208 2969/2556
Email: tgraju@jncasr.ac.in

Supplementary Schemes, Figures and Tables

Synthesis

Starting precursors **3** and **5** were prepared as follows[1, 2]

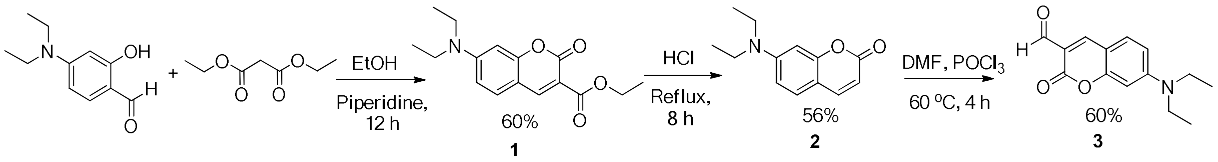

Scheme S1. Synthesis of compound **3**

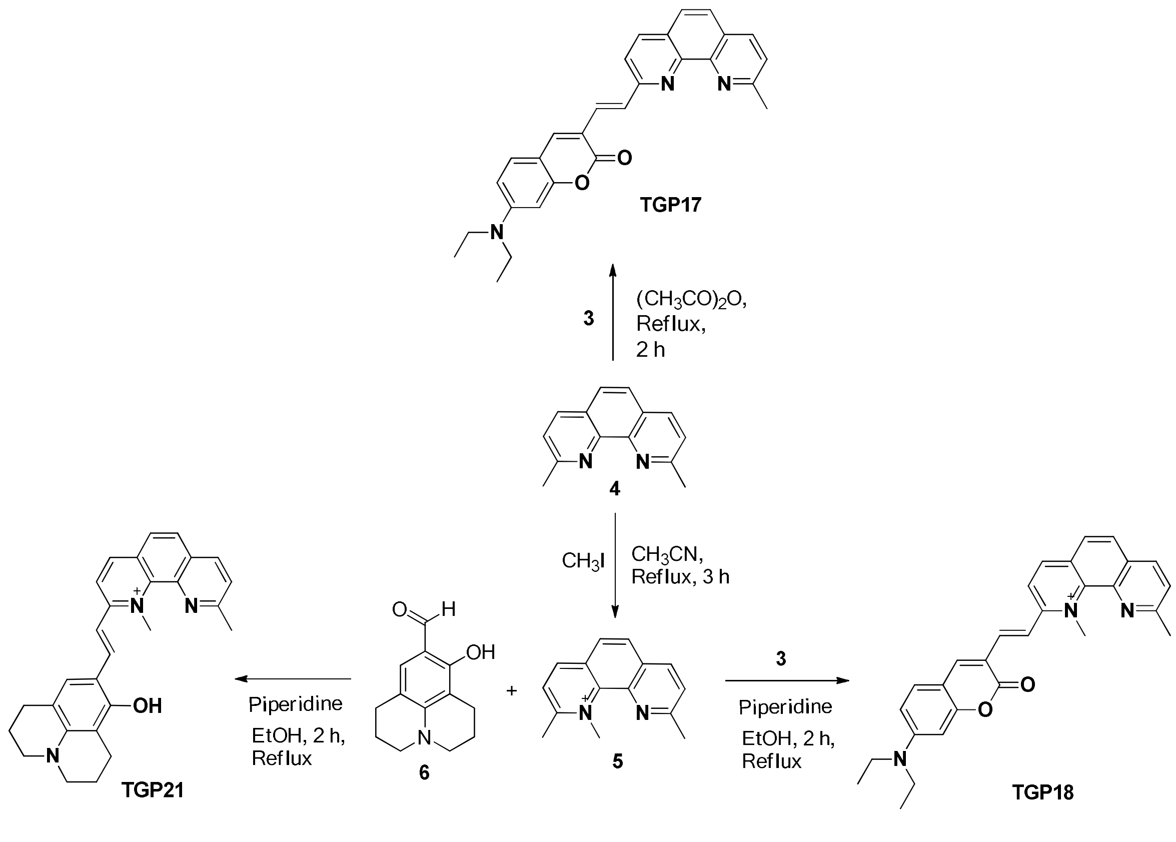

Scheme S2. Synthetic routes to neocuproine derivatives (TGP17, TGP18 and TGP21).

Compound characterization

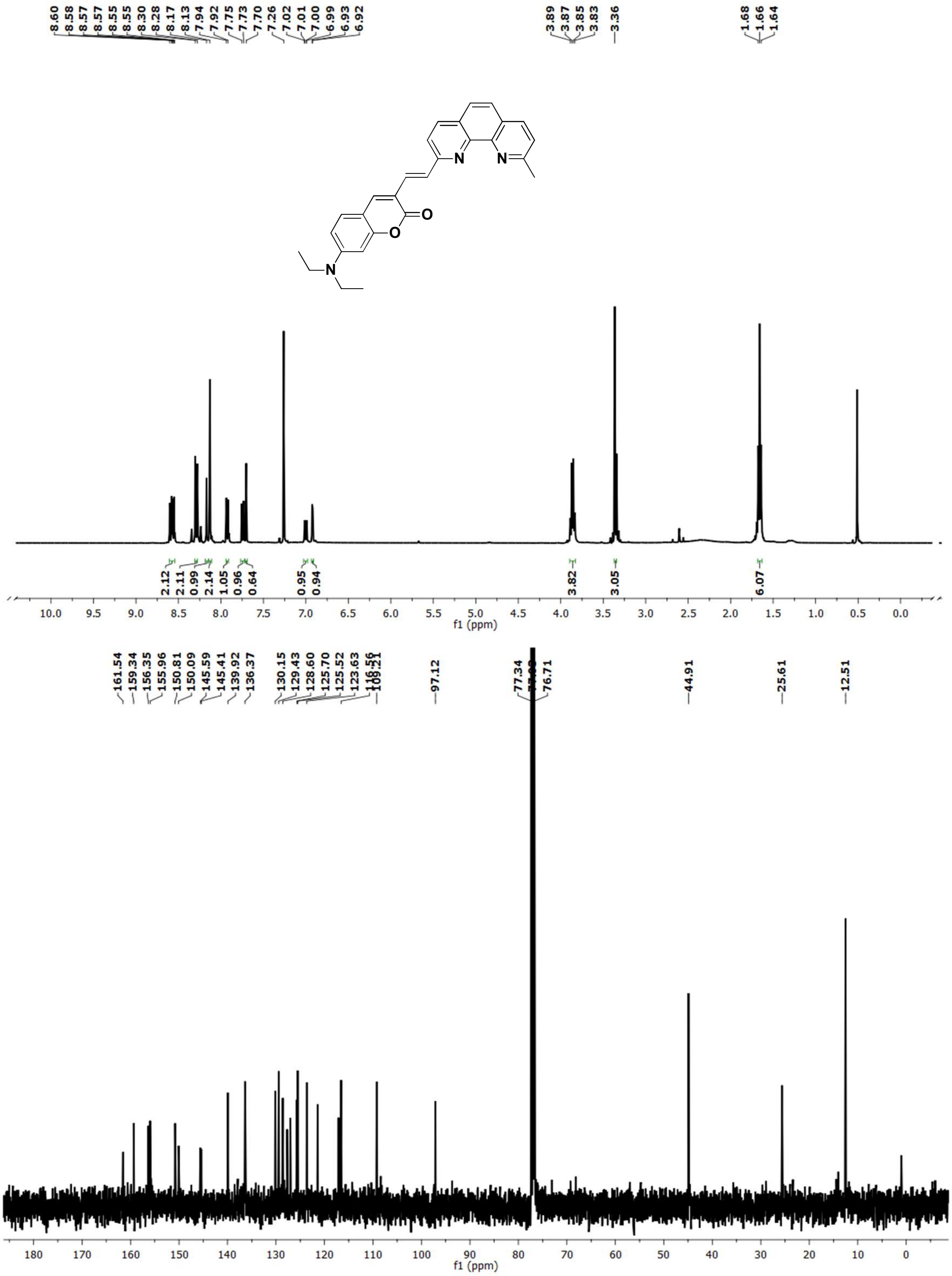

Figure S1. ^{1}H and ^{13}C NMR spectra of TGP17

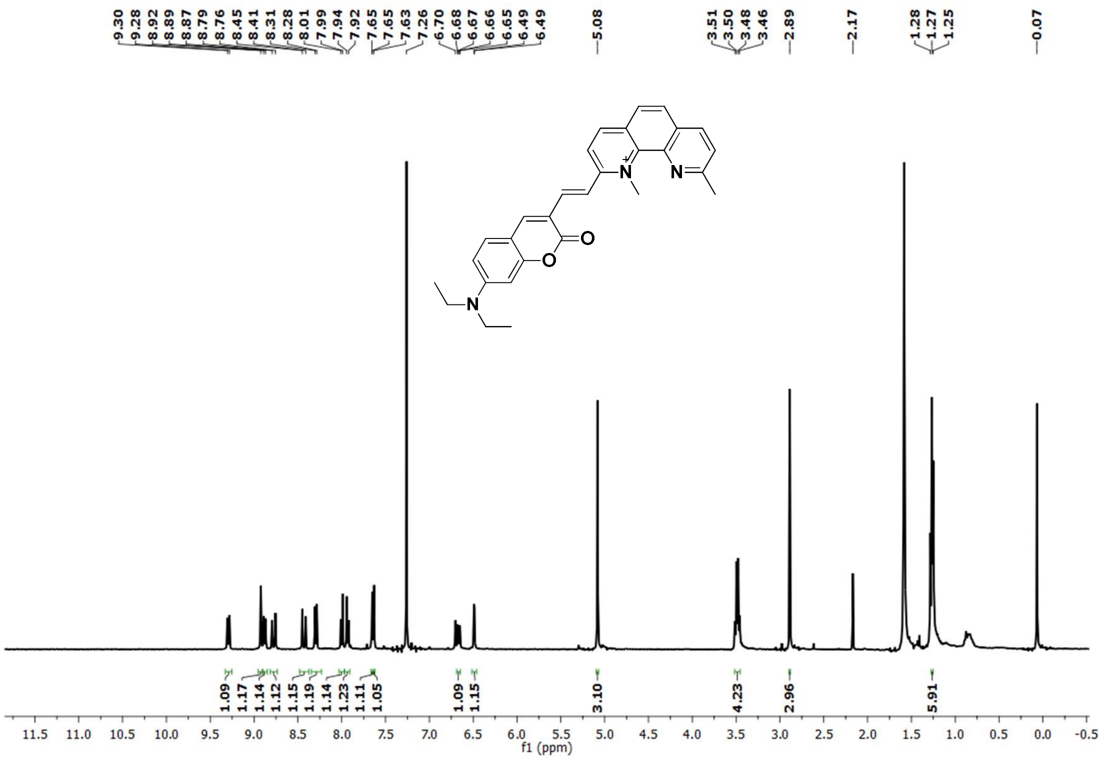

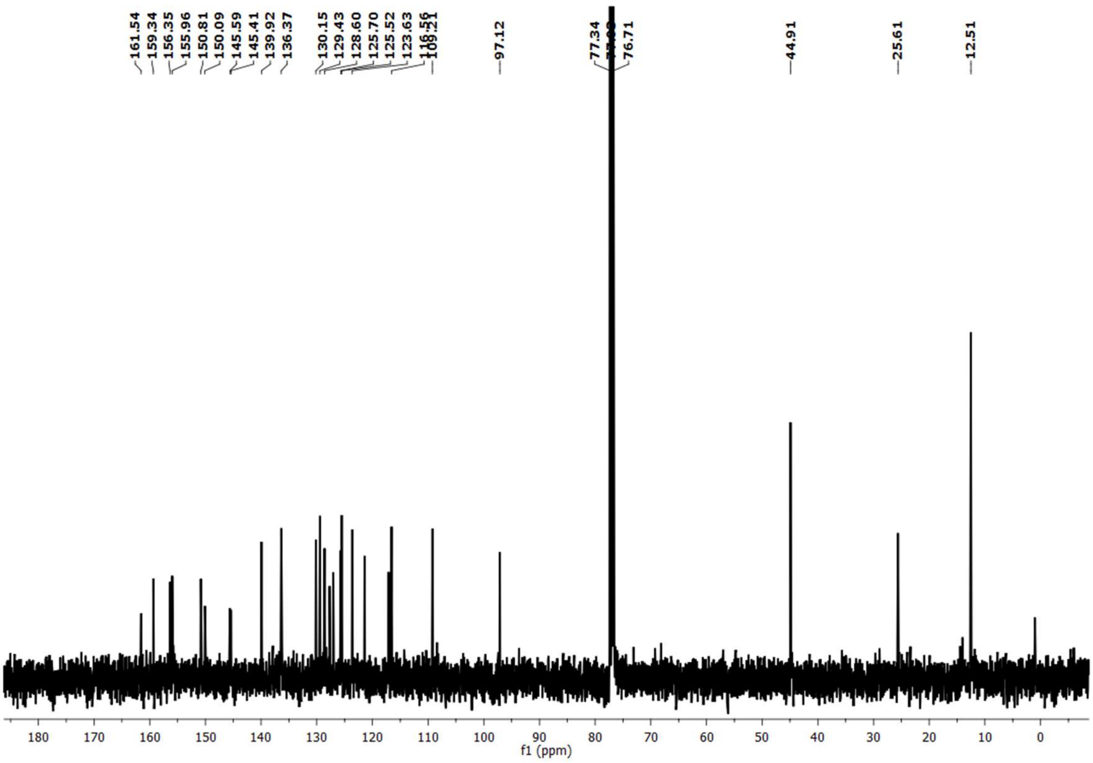

Figure S2. ^{1}H and ^{13}C NMR spectra of TGP18

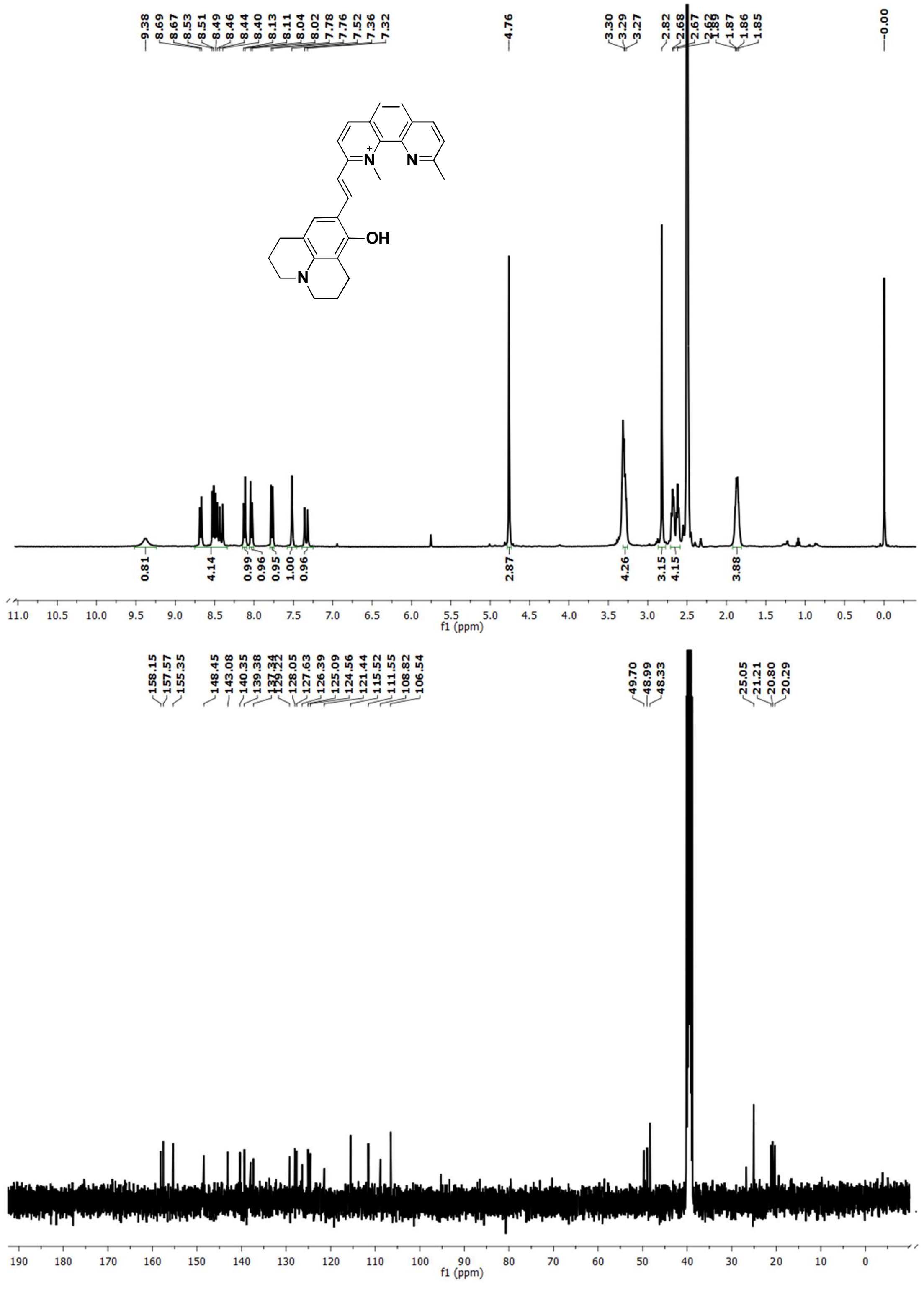

Figure S3. ^{1}H and ^{13}C NMR spectra of TGP21

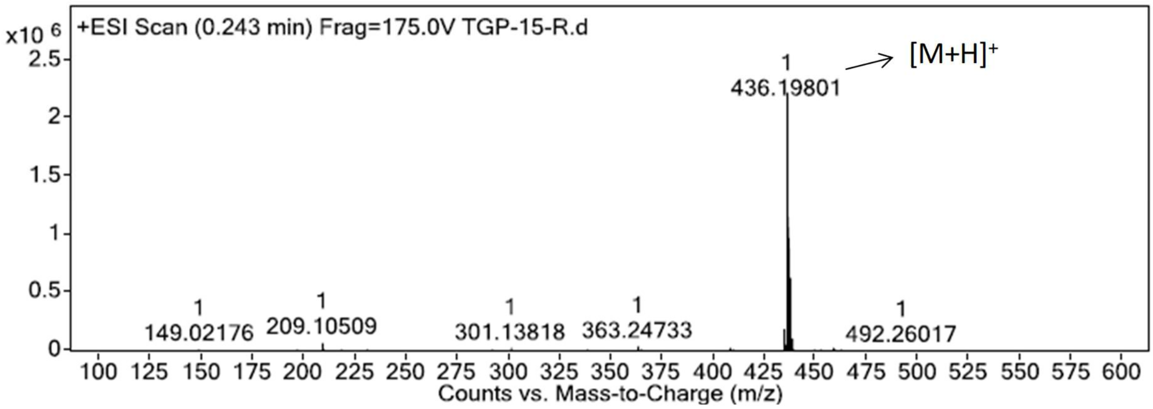

Figure S4. HRMS of TGP17 (Exact mass: 435.1947)

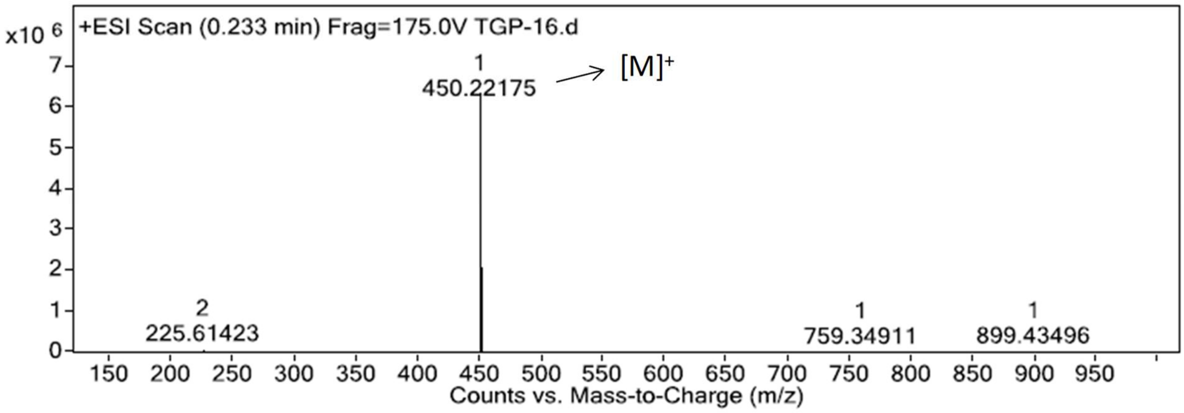

Figure S5. HRMS of TGP18 (Exact mass: 450.2182)

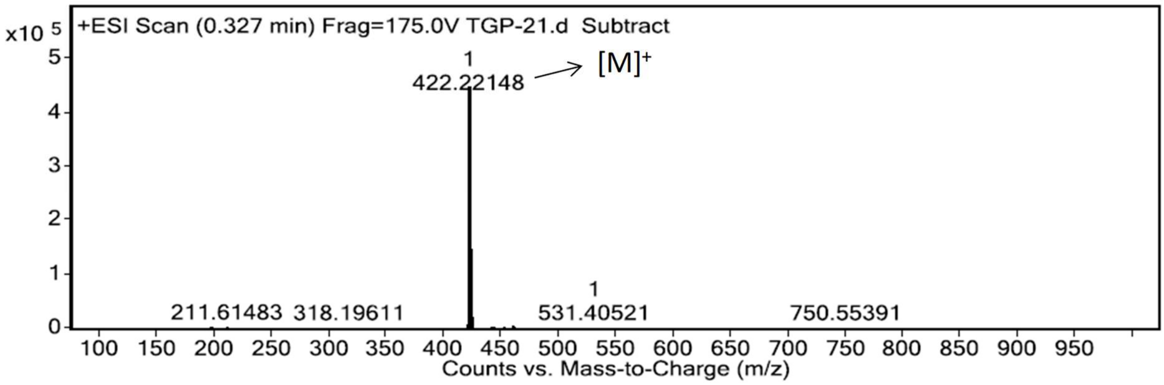

Figure S6. HRMS of TGP21 (Exact mass: 422.22148)

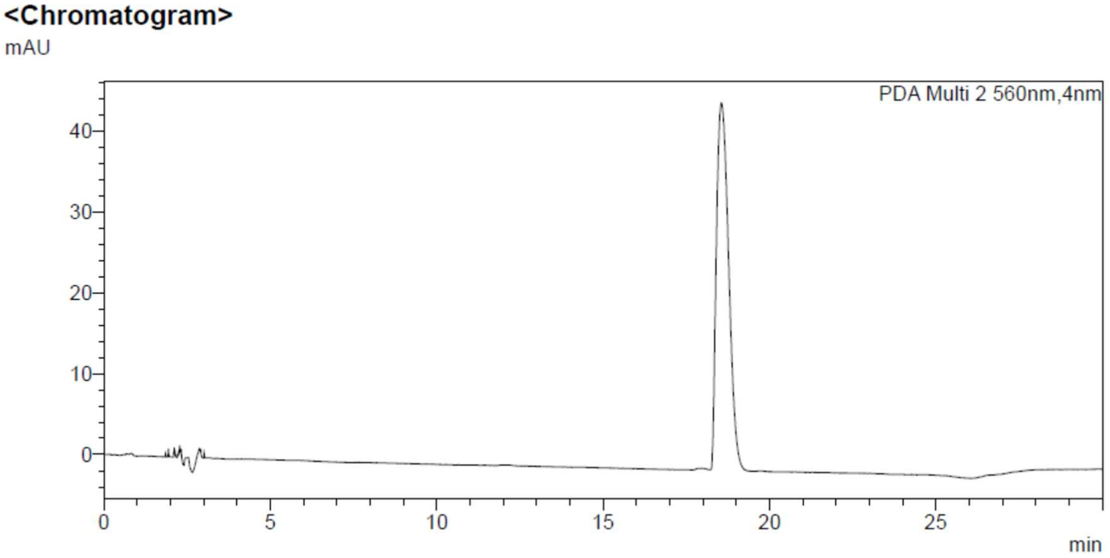

Figure S7. HPLC chromatogram of TGP18

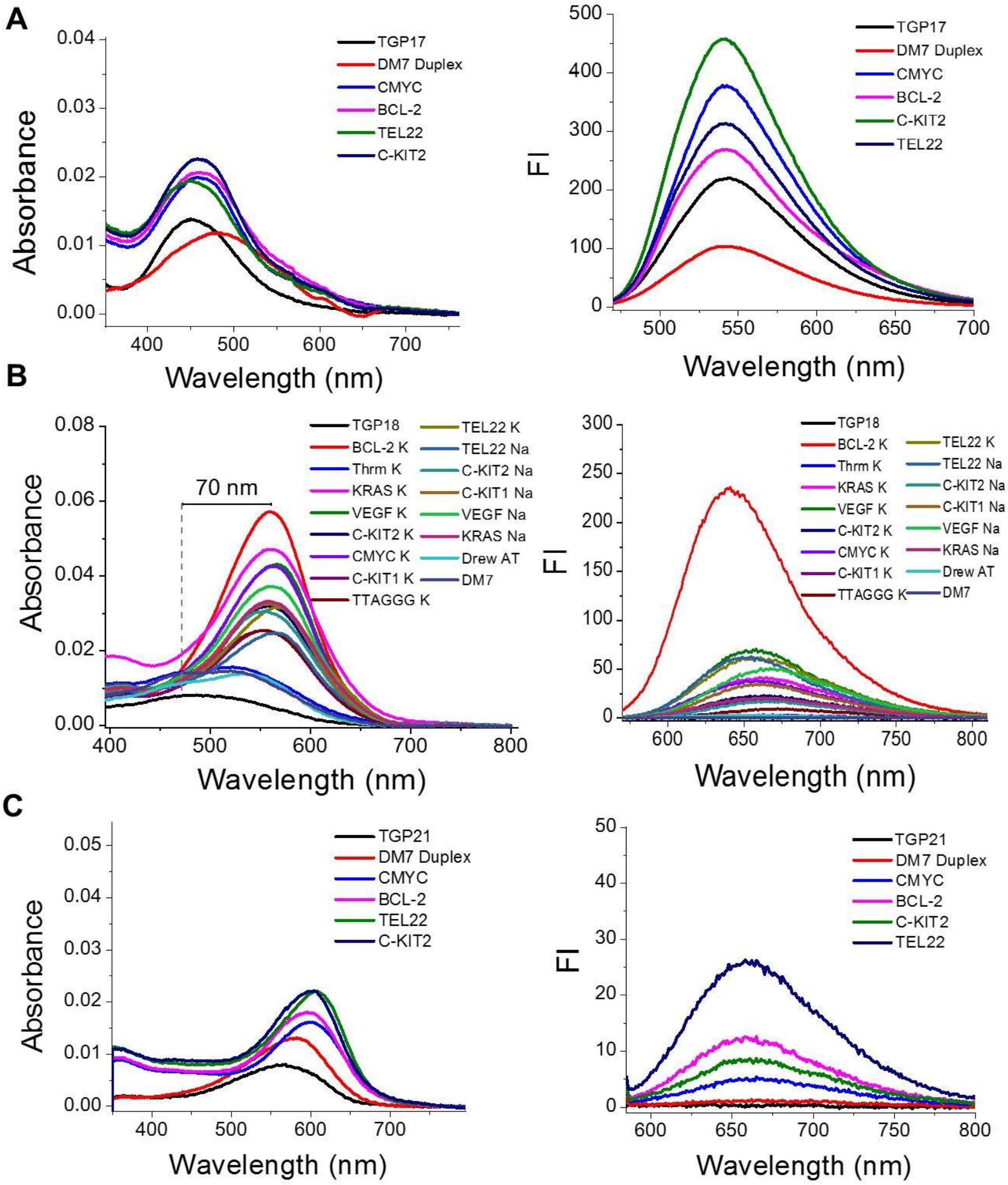

Figure S8. Photophysical measurements of neocuproine conjugates. Absorption and emission spectra of (A) TGP17, (B) TGP18 and (C) TGP21 in presence and absence of GQs and duplex DNA recorded in 20 mM phosphate buffered saline containing 100 mM KCl (pH 7.4). FI: fluorescence intensity.

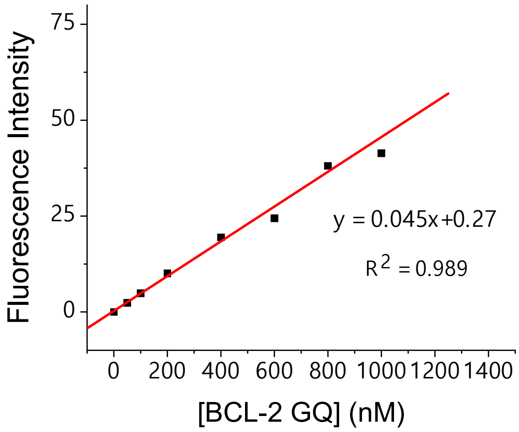

Figure S9. Determination of LOD. The plot of fluorescent intensity of TGP18 (2 μM) at 640 nm upon titrating against varying concentration of BCL-2 (0–1000 nM) in phosphate buffered saline (20 mM PBS, 100 mM KCl, pH 7.4). The data was fitted by linear equation to calculate LOD of TGP18 for BCL-2 GQ.

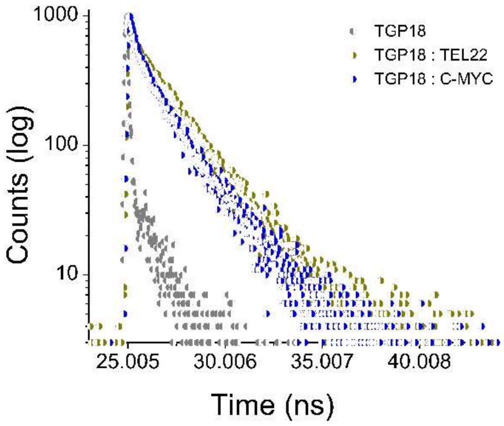

Figure S10. Fluorescence lifetime measurements of TGP18 alone and in presence of TEL22 and C-MYC GQs.

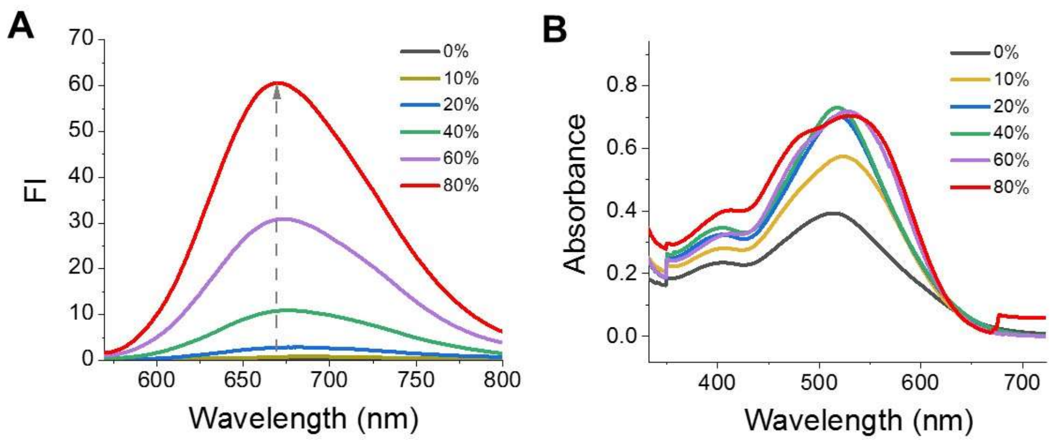

Figure S11. Viscosity dependent fluorescence and absorption spectra of TGP18. (A) Fluorescence and (B) absorbance spectra of TGP18 in phosphate buffered saline (20 mM PBS, 100 mM KCl, pH 7.4) with increasing glycerol percentage from 0 to 80%. FI: Fluorescence intensity.

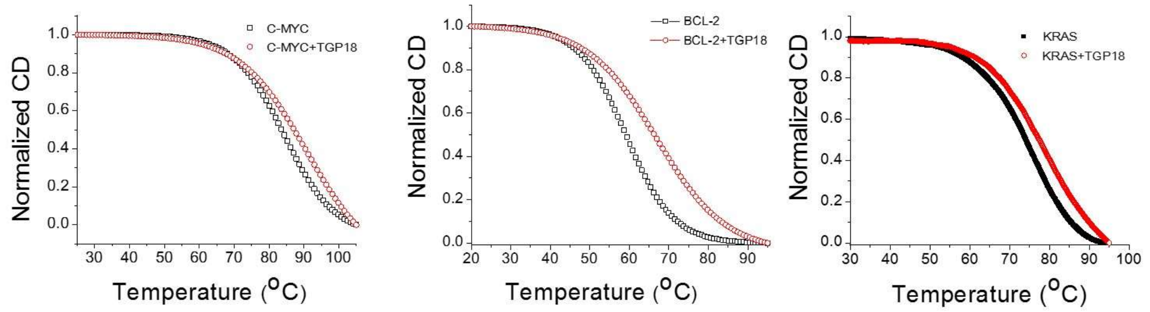

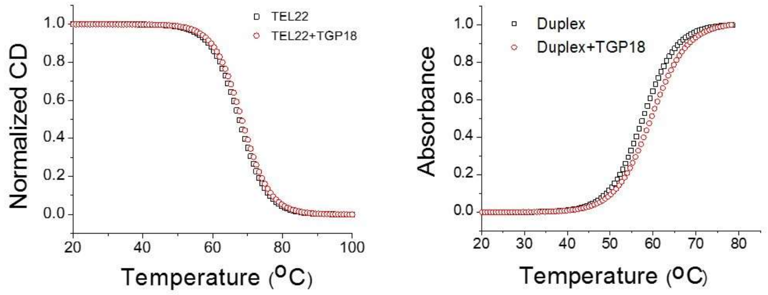

GQ+TGP18	T_m_GQ+TGP18 (°C)	ΔT_m (°C)
C-MYC	90.4	4.9
BCL-2	67.8	8.0
KRAS	79.0	3.5
TEL22	68.4	0.8
Duplex	61.2	1.2

Figure S12. Thermal melting profile of different GQ and duplex DNA sequences, and its complex with TGP18 (in ratio 1:1). Thermal stabilization (ΔT_m) for GQs and duplex are given in the table.

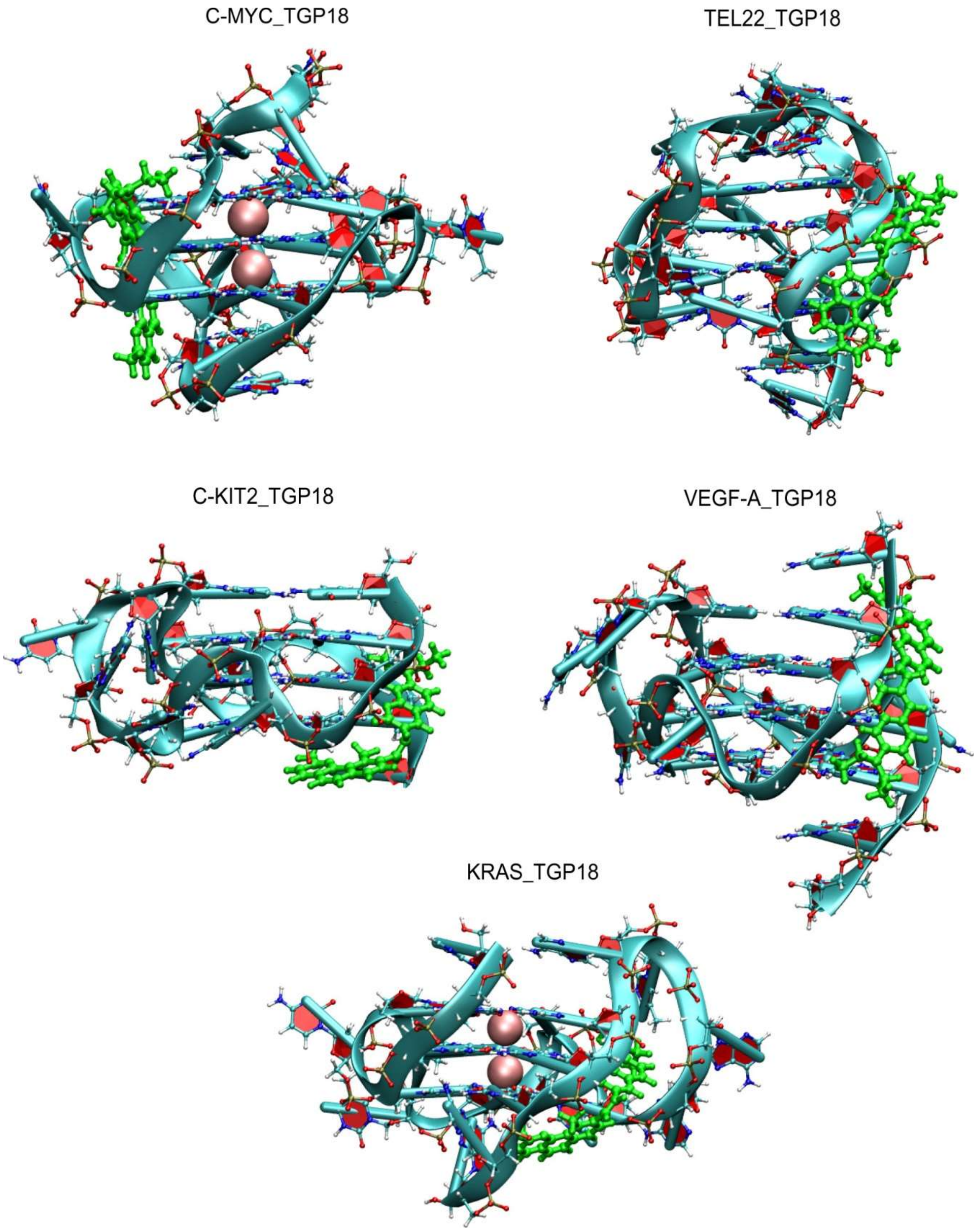

Figure S13. The binding modes of TGP18 (green) to other oncogene GQs (C-MYC, TEL22, C-KIT2, VEGF-A and KRAS) in the groove regions.

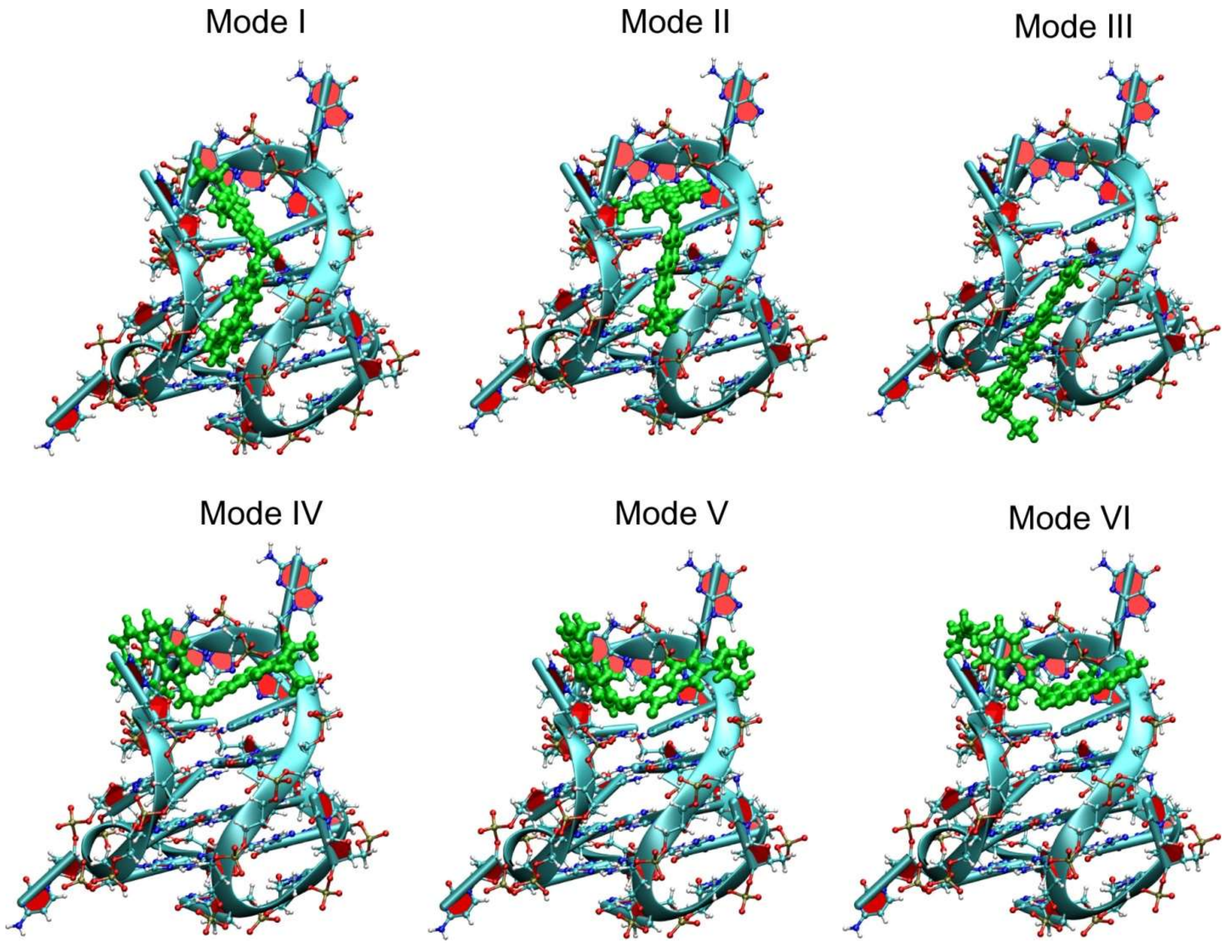

Figure S14. Six different binding modes of TGP18 (green) to BCL-2 GQ. Three loop-stacking modes (bottom panel: Mode IV, V & VI) and remaining groove binding modes (top panel: Mode I, II & III).

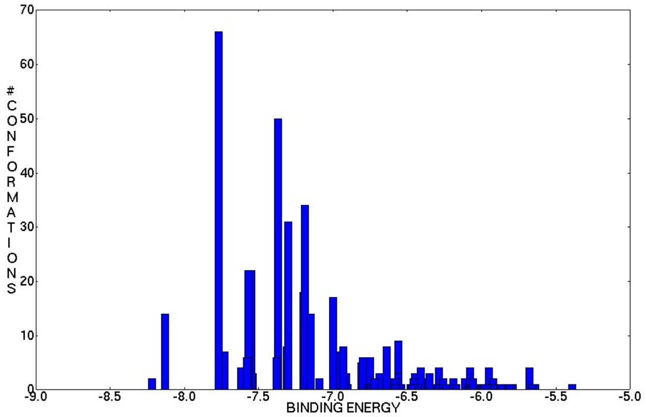

Figure S15. Multiple binding modes observed for TGP18 with BCL-2 GQ. For the subsequent molecular dynamics simulations only the independent and high affinity binding modes of TGP18 were considered.

A

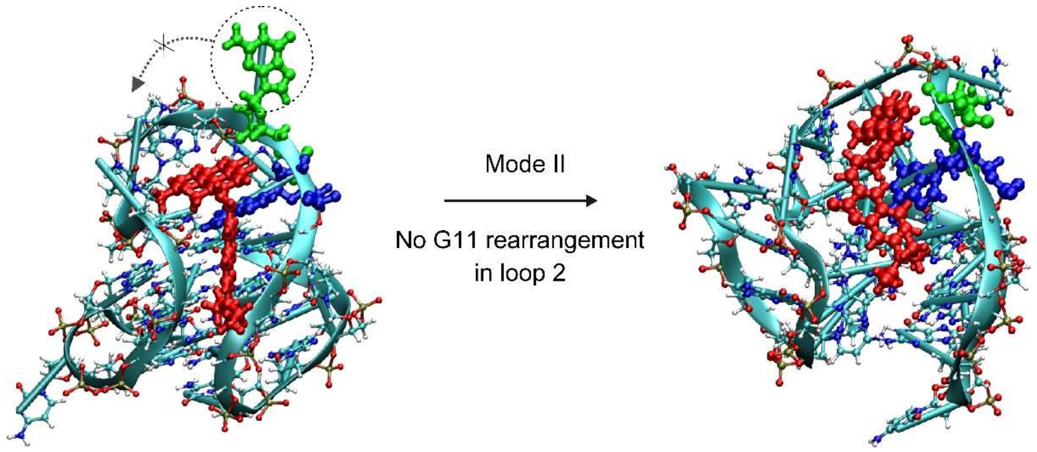

B

	Interacting moiety of TGP18	Interacting nucleobase	Mode of interaction	Bond distance (A°)	Binding free energy
Mode II	Neocuproine	A10	π–π	3.90	-35.5 kcal/mol
	Neocuproine_H17	G17	Carbon H-bond	2.31	
	Coumarin	G17	π–π	3.74	
	Neocuproine_C10	A10	π–alkyl	3.77	
	Neocuproine_C10	A14	π–alkyl	3.89	
	Neocuproine_C10	T15	π–alkyl	4.21	
	Coumarin_C29	G17	π–alkyl	5.40	

Figure S16. Visualizations and molecular interactions of TGP18 with BCL-2 GQ in Mode II. (A) Representative docked and simulated (50 ns long) configurations for BCL-

2:TGP18 Mode II complex showing absence of G11 (green) rearrangement in loop-2. Mode II shows hybrid binding involving neocuproine moiety stacked with A10 (blue) alone for loop-stacking and coumarin in groove binding interactions. (B) Summary of molecular interactions between TGP18 and BCL-2 in Mode II binding.

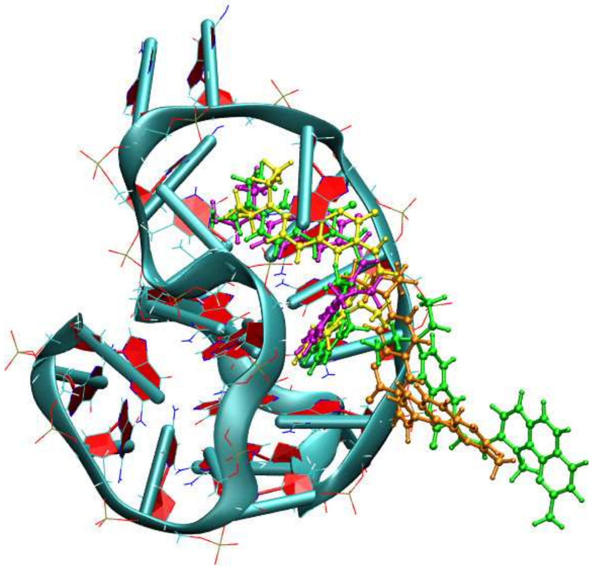

Figure S17. Egression (unbinding) pathway for binding Mode I of TGP18 with BCL-2 GQ from its bound state as predicted from RAMD simulation.

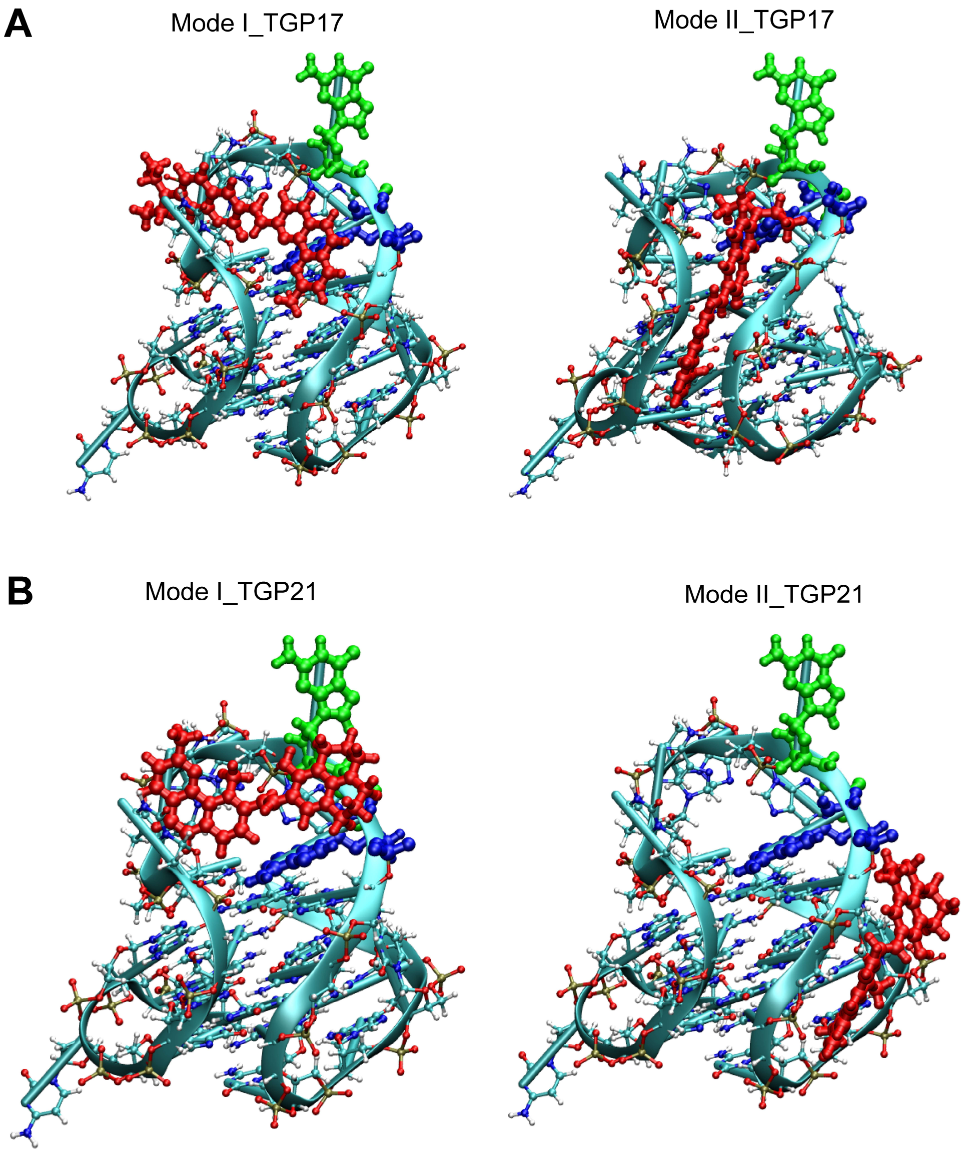

Figure S18. Visualization of TGP17 and TGP21 interactions with BCL-2 GQ. High affinity binding modes of (A) TGP17 (red) and (B) TGP21 (red) with BCL-2 GQ. As can be seen, loop-stacking (Mode I) on left side and groove binding (Mode II) on right side.

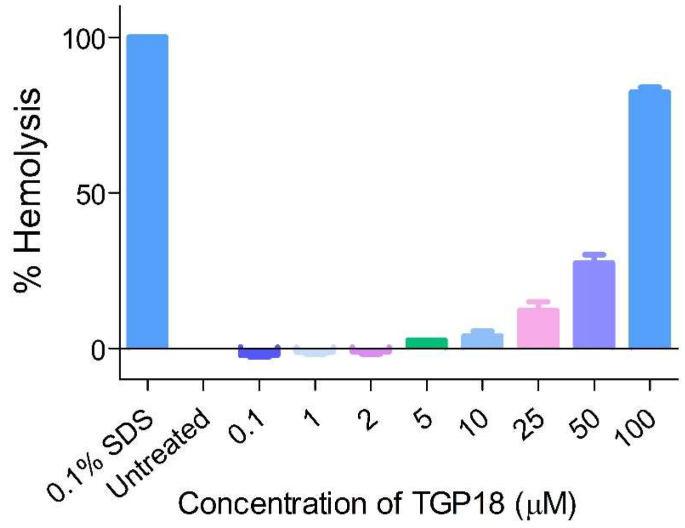

Figure S19. Effect of TGP18 on hemolysis of red blood cells (in %).

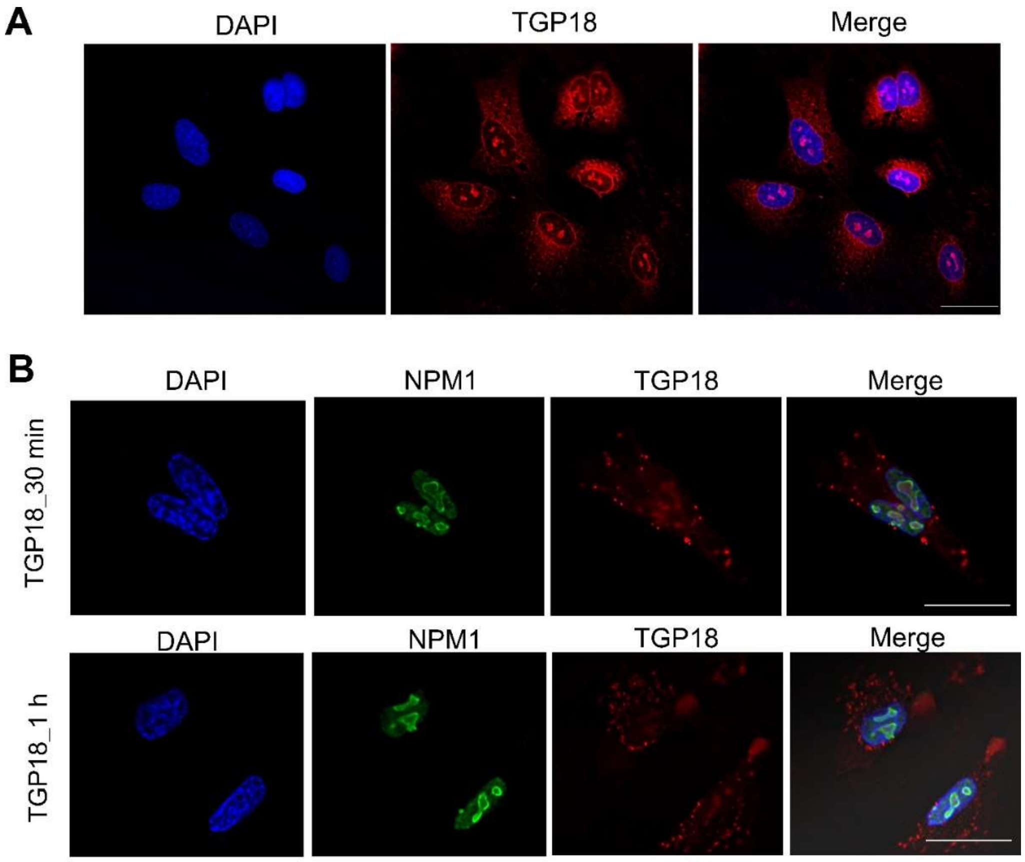

Figure S20. TGP18 localization in A549 cells. (A) TGP18 localization in fixed A549 cells. (B) Immunofluorescence images showing colocalization of NPM1 (nucleophosmin 1, green) with TGP18 (red). A549 cells were treated with 1 μM TGP18 (red) for 30 min and 1 h. Scale bar 25 μm.

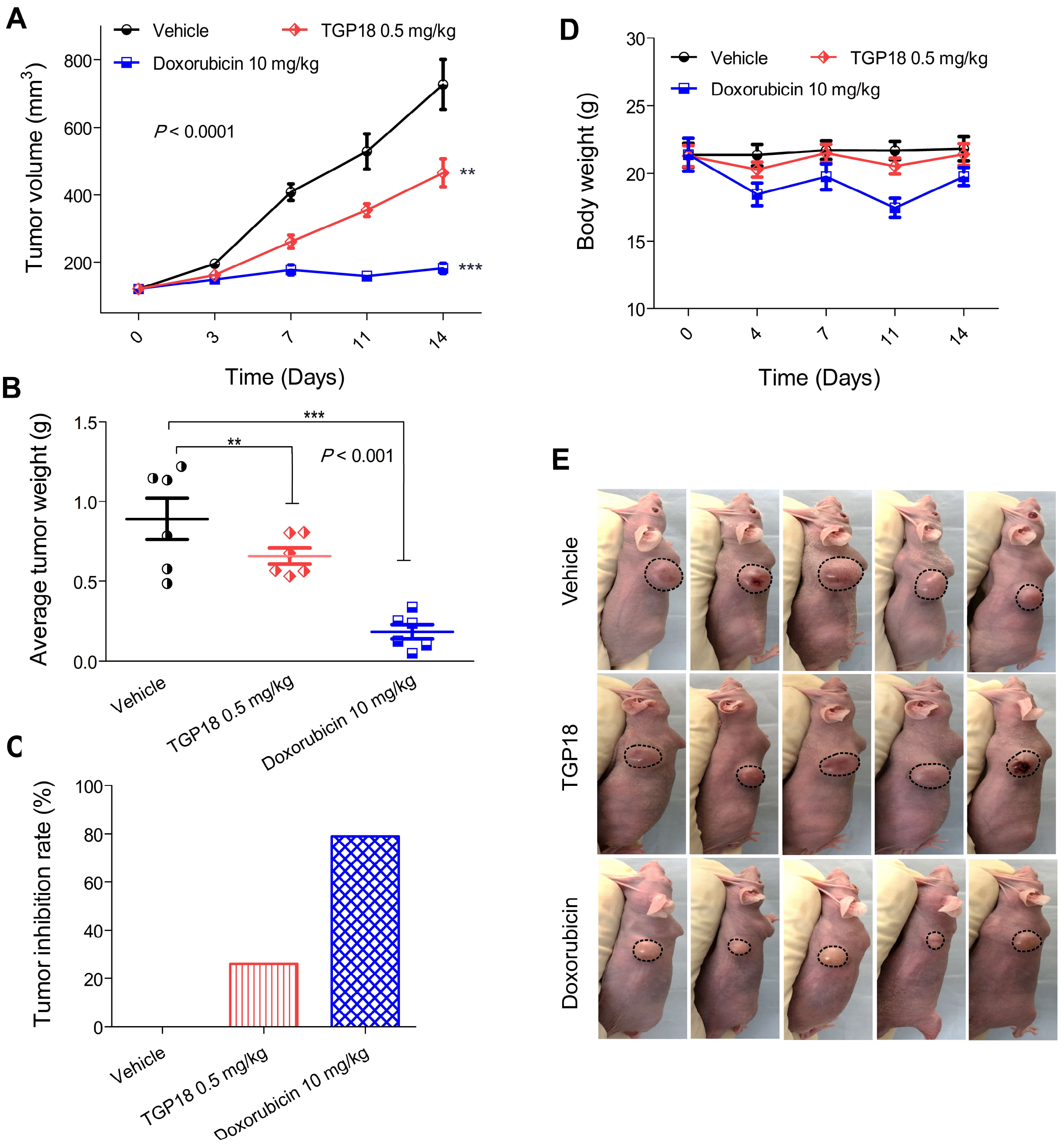

Figure S21. Efficacy of TGP18 in subcutaneously treated MDA-MB-231 tumor xenografts. (A) Tumor growth suppression in mice (n = 6) treated with TGP18 and doxorubicin compared with vehicle treated mice. (B) Average tumor weight and (C) tumor inhibition rate of mice (n = 6) for the three experimental groups monitored after 14 days treatment. *P* values were determined by one-way ANOVA followed by Dunnett's test for treated compared to control (vehicle). (D) Body weight monitored over treatment time for the three experimental groups of mice. (E) Representative images of mouse

bearing MDA-MB-231 xenografts following the treatment with either vehicle or TGP18 (0.5 mg/kg) or doxorubicin (10 mg/kg) for two weeks.

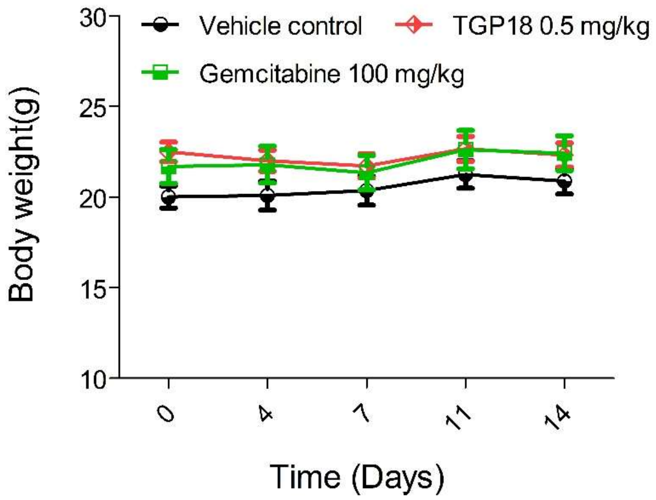

Figure S22. Body weights of mice bearing lung cancer for vehicle (black), TGP18 (red) and gemcitabine (green) treatment over time for the three experimental groups of mice.

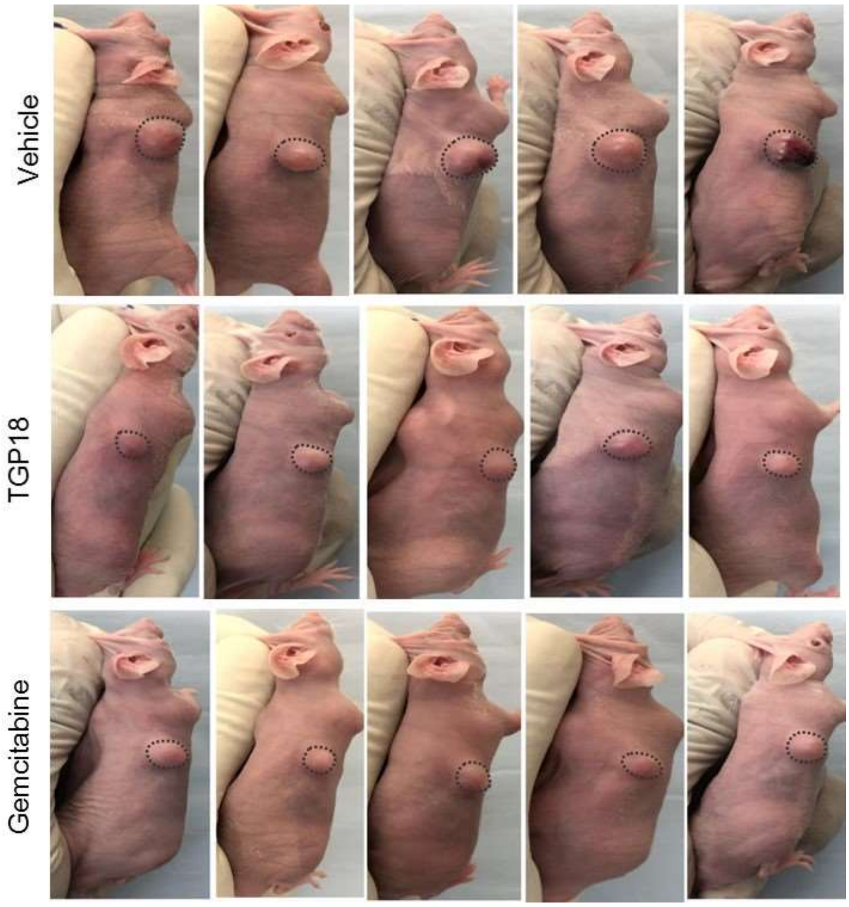

Figure S23. Representative images of mice bearing A549 xenografts following the treatment with either vehicle or TGP18 (0.5 mg/kg) or gemcitabine (100 mg/kg) for two weeks.

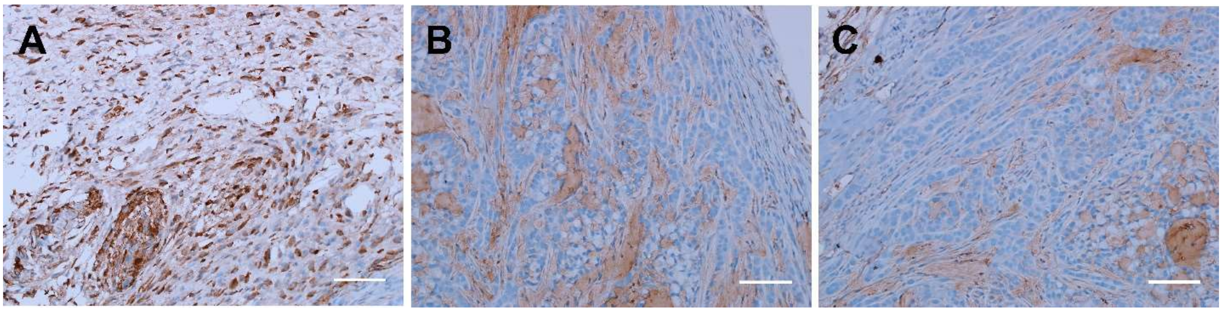

Figure S24. Immunohistochemical staining of BCL-2 gene in A549 tumor sections treated with (A) Vehicle control (B) TGP18 and (C) Gemcitabine. Scale bar 100 μm.

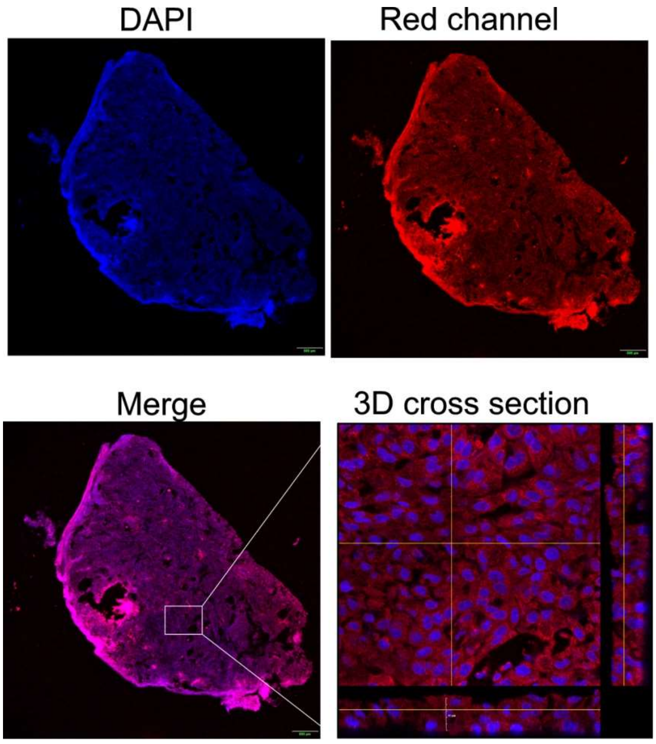

Figure S25. TGP18 penetration in A549 xenograft sections. 3D cross sectioning of tumor sections close to the tumor surface of A549 xenografts treated with TGP18 followed by nuclear staining with DAPI. The fluorescence images were obtained in the blue channel (DAPI) and red channel (TGP18).

Table S1. GQ forming oligonucleotide sequences located in the promoters of oncogenes (BCL-2, VEGF, C-MYC, KRAS, and C-KIT), Thrombin, and Telomere (TEL22), and duplex forming sequences studied.

	**Promoter**	**Sequence 5'-3'**
GQ forming sequences	BCL-2	CGGGCGCGGGAGGAATTGGGCGGGAGC
	VEGF	GGGGGCGGGCCGGGGGCGGGGTCCCGGCGGGGCGGAG
	KRAS	GGGAGGGAGGGAAGGAGGGAGGGAGGGA
	C-MYC	TGGGGAGGGTGGGGAGGGTGGGGA AGG
	BCL-2a	GGGCGCGGGAGGAAGGGGGCGGG
	TEL22	AGGGTTAGGGTTAGGGTTAGGG
	C-KIT2	CGGGCGGGCGCGAGGGAGGGG
	C-KIT1	AGGGAGGGCGCTGGGAGGAGGG
	Thrombin	GGGTTGGTGTGGTTGGA
Duplex forming sequences	DrewAT	GCGCAAATTTGCGC
	DM7	GCGCGAATTCGCGC

Table S2. Primer sequences used in qRT-PCR

**Primer**	**Sequence 5'-3'**
GAPDH-F	GATGCTGGCGCTGAGTACGTCGTG
GAPDH-R	AGTGATGGCATGGACTGTGGTCATGAG
KRAS-F	GACGAATATGATCCAACAATAGAGGATTC
KRAS-R	TAGGTACATCTTCAGAGTCCTTAACTC
VEGFA-F	GGGCAGAATCATCACGAAGTGGTG
VEGFA-R	CTGCATGGTGATGTTGGACTCCTCA
BCL-2-F	GTCATGTGTGTGGAGAGCGTCAACC
BCL-2-R	CCAGGGCCAAACTGAGCAGAGTC
C-MYC-F	TGAGGAGACACCGCCCAC
C-MYC-R	CAACATCGATTTCTTCCTCATCTTC

Table S3. Free energy of binding of TGP18 to different oncogene specific GQ structures/conformations. The free energy of binding of TGP18 with different GQswere computed from molecular dynamics trajectories by employing MM-GBSA approach. The unit is kcal/mol.

G-quadruplex	E_vdw	E_elec	E_polar	E_nonpolar	ΔG	Mode of binding
BCL-2 (PDB ID: 2F8U)	-61.5	-570.3	594.7	-5.0	-42.2	Hybrid
C-MYC (PDB ID: 1XAV)	-46.6	-510.8	540.0	-4.1	-21.5	Groove
TEL22 (PDB ID: 2HY9)	-30.2	-509.9	528.9	-3.2	-14.5	Groove
C-KIT2 (PDB ID: 2KYP)	-34.8	-446.2	462.1	-2.6	-21.5	Groove
VEGF-A (PDB ID: 2M27)	-28.5	-486.5	494.6	-2.0	-22.4	Groove
KRAS (PDB ID: 5I2V)	-56.3	-519.6	545.9	-4.8	-34.8	Groove

Table S4. The binding free energies computed for the six different modes (surface and minor groove binding) of TGP18 with BCL-2 GQ. The energies are in kcal/mol.

Mode	ΔE_{vdw}	ΔE_{elec}	ΔE_psolv	ΔE_npsolv	ΔG
Mode I	-61.5	-570.3	594.7	-5.0	-42.2
Mode II	-47.0	-520.8	536.4	-3.6	-35.0
Mode III	-34.7	-464.2	477.5	-2.6	-24.0
Mode IV	-42.5	-499.8	520.9	-3.4	-24.8
Mode V	-41.5	-469.3	487.5	-2.9	-26.2
Mode VI	-37.1	-485.2	503.4	-3.3	-22.3

Table S5. The free energy of Mode I binding computed for TGP18 along the egression pathway as predicted from RAMD simulations. The configurations along the binding pathway were used in the MM-GBSA calculations. The results are only presented for binding Mode I. The energies are in kcal/mol.

Mode	ΔE_{vdw}	ΔE_{elec}	ΔE_psolv	ΔE_npsolv	ΔG
State-1	-60.3	-568.2	593.4	-4.9	-40.1
State-2	-51.9	-549.4	572.5	-4.6	-33.3
State-3	-49.4	-535.6	558.7	-4.5	-30.8
State-4	-39.5	-506.1	526.0	-3.7	-23.3
State-5	-15.9	-411.1	423.4	-1.8	-5.4

Table S6. The binding free energies computed for the two modes (loop-stacking and groove binding) of TGP17 and TGP21 with BCL-2 GQ. The energies are in kcal/mol.

Mode	ΔE_{vdw}	ΔE_{elec}	ΔE_psolv	ΔE_npsolv	ΔG	Mode of binding
		**TGP17**				
Mode I	-55.0	54.1	-18.2	-4.7	-23.8	Loop-stacking
Mode II	-26.1	17.1	3.6	-2.6	-8.0	Groove
		**TGP21**				
Mode I	-40.0	-511.7	530.6	-3.5	-24.6	Loop-stacking
Mode II	-34.3	-490.6	508.1	-2.6	-19.4	Groove

References

1. Maity D, Govindaraju T. Highly selective colorimetric chemosensor for Co^{2+}. Inorg Chem. 2011; 50: 11282-4.

2. Li B, Qin X, You J, Cong X, Lan J. Direct arylation of phenanthroline derivatives via oxidative C–H/C–H cross-coupling: synthesis and discovery of excellent ligands. Org Biomol Chem. 2013; 11: 1290-3.
